# Grape Stalks Valorization towards Circular Economy: A Cascade Biorefinery Strategy

**DOI:** 10.1002/cssc.202402536

**Published:** 2025-02-14

**Authors:** Carlotta Valle, Giorgio Grillo, Emanuela Calcio Gaudino, Paola Ponsetto, Roberto Mazzoli, Giulia Bonavita, Pietro Vitale, Enrica Pessione, Emilia Garcia‐Moruno, Antonella Costantini, Giancarlo Cravotto, Silvia Tabasso

**Affiliations:** ^1^ Department of Drug Science and Technology University of Turin Via P. Giuria 9 10125 Turin Italy; ^2^ Department of Life Sciences and Systems Biology University of Turin Via Accademia Albertina 13 10123 Turin Italy; ^3^ Research Centre for Viticulture and Enology CREA-VE Council of Agricultural Research and Economics Via Pietro Micca 35 14100 Asti Italy

**Keywords:** integrated biorefinery, lactic acid, microwave chemistry, subcritical water• sustainable chemistry

## Abstract

Lignocellulosic biomasses have the potential to generate by‐products with biological activity (i. e., polyphenols) as well as biopolymers (i. e., cellulose, hemicellulose, pectins, lignin). The wine industry is one of the pillars of Italian agri‐food sector. Nevertheless, large quantities of by‐products such as grape stems are produced, which are usually disposed of at a cost, and therefore represent an attractive negative‐cost feedstock for biorefinery. In this work, a sequential protocol for biomass valorization is proposed, characterized by a multidisciplinary strategy using enabling technologies and subcritical water as a green solvent, where physical/chemical treatments synergistically interact with biological treatments. The first phase involved the sequential fractionation of grape stalks, obtaining several product streams rich in polyphenols, hemicellulose, pectin (13.15 % of cumulative yield on biomass), lignin and cellulose. A membrane treatment was employed to recycle materials within the process. Finally, the cellulose‐rich residue was exploited as a fermentation substrate for the last step, producing up to 5.8 g/L of lactic acid by harnessing suitably engineered *Clostridium thermocellum* strains. The polyphenolic fraction successfully inhibited the growth of *Brettanomyces bruxellensis* and *Acetobacter pasteurianus*, microorganisms responsible for major wine off‐flavors. Globally, this study represents a proof‐of‐concept of a second‐generation biorefining process based on locally available waste biomass.

## Graphical abstract

A cascade protocol for grape stalks valorization has been proposed, characterized by a multidisciplinary strategy using unconventional techniques and subcritical water as a green solvent, where physical/chemical treatments synergistically interact with biological treatments to obtain added value products from all the biomass components.

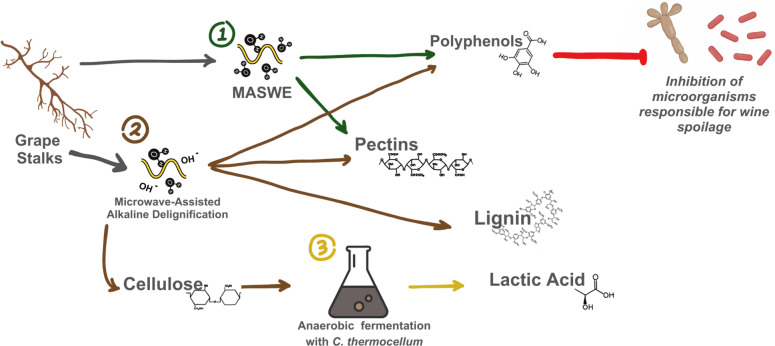

## Introduction

The concept of circular economy changes the current production and consumption model based on “take‐make‐dispose” managing resources in a closed‐loop.^[1]^ This concept allows the reuse of waste and helps to meet some of the seventeen sustainable developmental goals proposed by the United Nations, such as fighting world hunger and poverty, ensuring health, supply clean water and renewable energy, promote employment, protect terrestrial and underwater life.^[2]^

Agricultural by‐products can be fully valorized through a biorefinery approach, sometimes (but not necessarily) using living organisms such as bacteria, algae, yeasts, or filamentous fungi to transform and convert waste into valuable compounds.^[3]^ Among the best exploited waste resources for bioconversions are worth mentioning those derived from the sugar cane industry (water, bagasse, molasses),^[4]^ milk whey, which has been largely explored as valuable carbon source for ethanol production (by yeast and bacteria fermentation) during the last decades of the 20^th^ century,^[5]^ activated sludge wastewater^[6]^ and lignocellulosic biomasses.^[7]^ The latter also encompasses forestry and agri‐food waste which generally are recalcitrant to degradation due to the complex biochemical structure of their macromolecules, which require different enzymes to convert it into available nutrients for microbial‐based bioconversions.^[3]^ The main components of lignocellulosic biomass are cellulose, hemicellulose, and lignin. The latter is an aromatic biopolymer that can be biodegraded mainly by fungi, whose genome encodes genes for ligninolytic enzymes such as lignin peroxidases, laccases and other enzymes able to open the phenolic ring of such molecules.^[8]^ Hemicelluloses are complex heteropolysaccharides consisting of both hexoses and pentoses that only a limited number of microbial species can use as substrate.^[9]^ In traditional biorefining processes, cellulose undergoes enzymatic saccharification prior to fermentation.^[10]^ However, direct fermentation of cellulose by native or engineered cellulolytic microorganisms provides a means to significantly reduce the process cost.[^11^, ^12^] The purification of cellulose through the removal of other biomass fractions and the optimization of the extraction of bioactive compounds from agri‐food waste poses challenges that are currently generating a lot of interest, since these compounds have various applications and a high demand on the market.[^13^, ^14^] Another fraction that can be found in biomass is pectin, which is a D‐galacturonic acid‐rich polysaccharide that is often used as a thickening agent in both cosmetic and food industry but also finds application in the nutraceutical industry thanks to its prebiotic properties.^[15]^

Among agri‐food waste, those deriving from the wine industry are highly significant, given that 260 million hectoliters of wine are produced every year worldwide.^[16]^ Grape stalks (GS) represent an important waste of the wine industry, whose exploitation is yet to be optimized, as they have a significant polluting potential caused by a high organic matter content.^[17]^ GS are, however, an interesting carbon substrate to grow bacteria, being rich in cellulose, hemicellulose, and pectin; moreover, they possess additional high value compounds such as polyphenols, which are widely used antioxidants and have recently been reported to be active antimicrobial agents.^[18]^

To make the valorization of the different components of agri‐food waste competitive, green extraction protocols must be developed.

These protocols involve the combination of enabling technologies and green solvents, aiming to reduce the environmental impact, energy consumption and health hazards associated with the use of traditional organic solvents.^[19]^ Several processes have been developed for this purpose, including microwave‐assisted extraction (MAE) and microwave‐assisted subcritical water extraction (MASWE).^[20]^

MAE is widely known to significantly reduce extraction times compared to conventional techniques, meanwhile enhancing the extraction yields.^[21]^ The shorter process times enable the preservation from degradation of labile compounds; this benefit is even better when using water in subcritical conditions..[^22^, ^23^] Under subcritical conditions, water can dissolve several components of medium and low polarity due to changes in its properties as temperature and pressure increase.^[24]^ Systematic decreases in permittivity, viscosity and surface tension, and an increase in the diffusion coefficient make water behave similarly to hydro‐alcoholic mixtures.^[25]^ Furthermore, when SWE is performed under microwave irradiation, the dielectric heating favors the rapid attainment of subcritical conditions, with temperatures ranging between 100 °C and 374 °C under pressure.^[26]^ Microwave‐assisted Subcritical Water Extraction (MASWE) combines the advantages of subcritical water (dielectric constant suitable for the solubilization of polyphenolic compounds,^[27]^ low viscosity,^[28]^ and high mass transport^[29]^) with microwave technology, which ensures a fast and homogeneous heating of the sample.^[30]^

**Figure 1:**
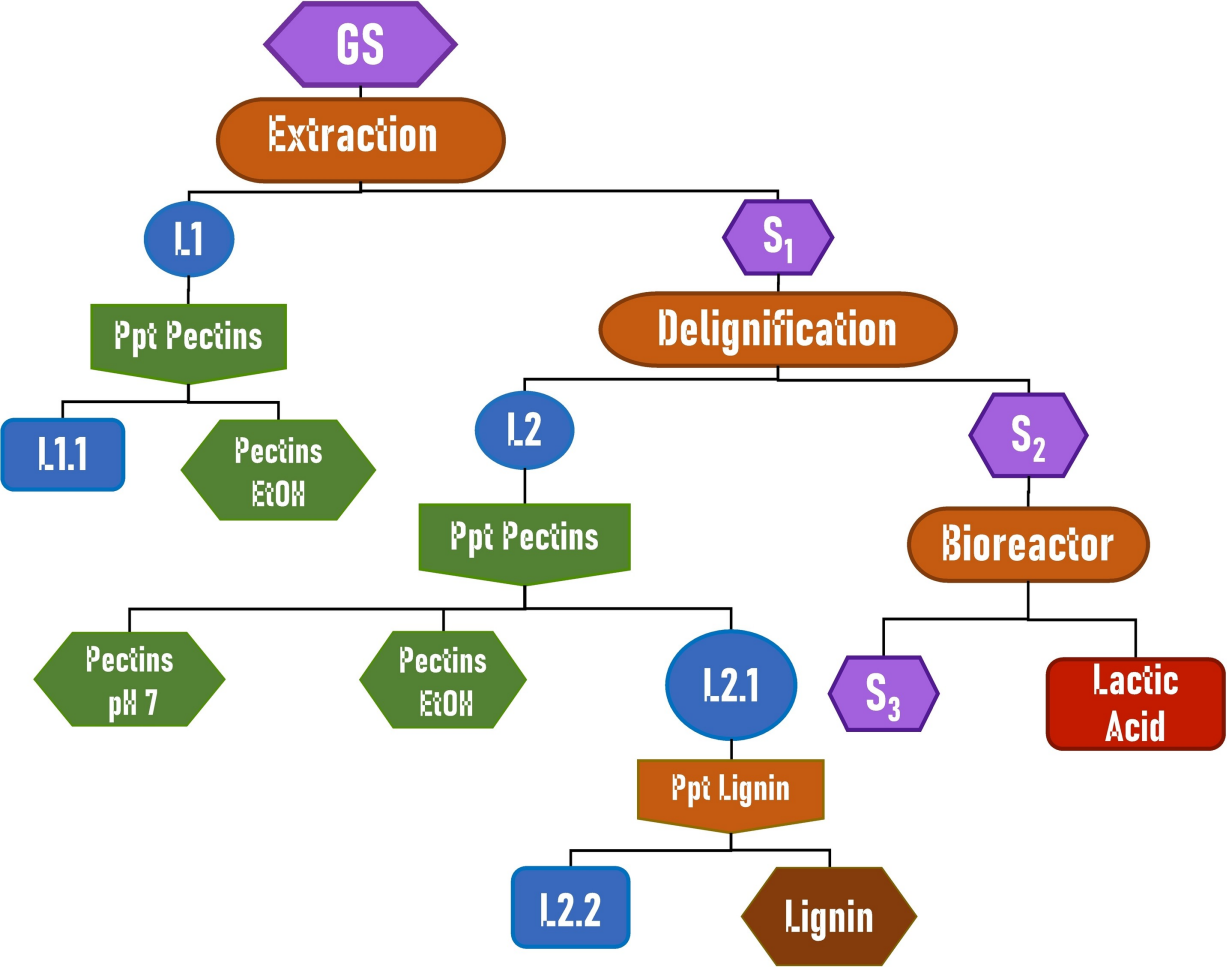
Cascade valorization protocol, schematic representation. L1: liquid fraction from MASWE; L1.1: L1 after pectins precipitations; S1: solid residue after MASWE; L2: liquid fraction after MW‐assisted delignification; L2.1: L2 after pectins precipitation; L2.2: L2.1 after acid‐insoluble lignin precipitation; S2: cellulose‐rich solid residue after delignification; S3: solid residue remaining after fermentation.

The present work aimed to develop a sustainable protocol for the chemical and biochemical valorization of all the components of GS, according to a biorefinery approach (Figure 1).

To this end, we developed a cascade process involving a MASWE of polyphenols, followed by an alkaline Microwave‐Assisted delignification, according to a two‐step‐one‐pot approach. The liquid fractions derived from these two steps contain mainly polyphenols, pectin and lignin.

The solid cellulose‐enriched fraction derived from the cascade process (S2) was indeed used as the main carbon source for two engineered L‐lactate‐overproducing *C. thermocellum* strains.

## Materials and Methods

### Biomass Feedstock

The GS of different cultivars from Piedmont region (Nebbiolo, Barbera, Dolcetto) were provided by CREA‐VE (Centro di Ricerca Viticoltura ed Enologia, Asti). Before being subjected to any treatment, the stalks were cryo‐milled (Waring Blender, HGBTWTS360, Stanford CT, USA) to increase the surface area of the martix.

### Benchmark Extraction

To compare the green extraction with well‐established conventional protocols, each cultivar was treated with EtOH: H_2_O (70/30) as solvent, with a solid‐to‐liquid ratio (w/v) of 1:30. The biomass was extracted for 1 h at 100 °C on a heated stirring plate. This procedure was conducted twice to ensure an exhaustive extraction. The resulting extract was freeze‐dried, weighed, and stored at 4 °C until further analyses.

### Cascade Protocol

**Extraction Step**. The MASWE on freeze‐dried GS was performed using a multimode microwave reactor (SynthWAVE, Milestone S.r.l., Bergamo, Italia) set at well‐defined conditions of temperature and inert gas pressure (N_2_), depending on the type of solvent used. Gas pressure was necessary to reach the subcritical state of water and avoid ebullition; the inert gas N_2_ was chosen to protect polyphenols from oxidation during the extraction process. The system was continuously stirred during the entire treatment. A chiller provided a fast cool‐down at the end of the protocol. The conditions screened using MASWE were 120 °C and 150 °C, for 7.5, 15 and 30 minutes.

The common conditions applied to the processes were the following: N_2_ pressure of 5 bar, temperature ramp of 5 minutes and a solid‐to‐liquid ratio of 1:15 g/mL. This ratio has been chosen in order to ensure a proper mixing of the matrix during the extraction and allowing an appropriate mass transfer in the pectin‐rich biomass. After the proper time of extraction, the extracts were separated by vacuum filtration and the resulting biomass was thoroughly washed. Both the liquid (L1) and the solid (S1) fractions were lyophilized, weighted, and stored at 4 °C before further analyses or treatments. Extraction Yield was calculated as g_Extr._/g_Matr._.

**Delignification Step**. The MW‐assisted delignification process involved the use of NaOH (10 % w/w on dry matter). The pH of the liquor was measured immediately after the addition of NaOH‐water solution to S1 (solid‐to liquid ratio 1:20 g/mL) and then after the delignification process (120 °C for 120 minutes, 5 bar N_2_[^31^, ^32^]) to quantify the extent to which NaOH reacted with the biomass during delignification. At the end of the treatment, the resulting mixture was then centrifuged and filtered. The solid biomass was washed until the pH of the supernatant was neutral. Both the liquid (L2) and the solid (S2) fractions were lyophilized, weighted, and stored at 4 °C before further analyses or treatments.

### Pectin/Lignin Isolation and Characterization

The solution resulting from the extraction process (L1) was treated with ethanol as anti‐solvent, to remove pectin by precipitation. Ethanol (96 %) was added in a 1:1 ratio with the liquid extract and stored at 4 °C for 2 h.^[33]^ The mixture was then centrifuged, and, after filtration, the pectic precipitate was washed with ethanol to remove any residual polyphenols. The supernatant (L1.1) was filtered and, before freeze‐drying, the ethanol was recovered through distillation. The remaining solid was lyophilized and weighted. Samples were stored at 4 °C before further analysis.

The solution resulting from the delignification process (L2) was treated with H_2_SO_4_ (96 %) and ethanol to induce the selective precipitation of specific compounds of interest. Briefly, after the neutralization of L2 with H_2_SO_4_ (96 %), which caused the first pectin fraction precipitation (Figure 1, pH 7), a second fraction (pectin EtOH) was isolated by means of the same treatment as reported for L1 (1:1 addition of ethanol).

After the precipitation of pectin, the remaining supernatant L2.1 was acidified with H_2_SO_4_ (96 %) until it reached pH 2 to precipitate acid‐insoluble lignin. Lignin was then separated from L2.2 (containing lignin‐derived polyphenols) through centrifugation and washed until neutrality with deionized water. Before each step a fraction of the liquid fraction was kept for further analysis. Resultant solid and liquid fractions were neutralized through acid/base addition or washing, respectively; subsequently, the fractions were lyophilized and weighted. Samples were stored at 4 °C before further analysis.

### Spectrophotometric Characterization of Extracts

**Total Phenolic Content (TPC) –** ***Folin‐Ciocalteu*** **Assay**. This analytical technique provides an indication of the selectivity and the yield of polyphenols of the antioxidant extracts.^[34]^ The absorbance values of the samples at 725 nm are compared to a calibration curve of gallic acid, employed as a reference in the analysis. Total polyphenols are calculated as equivalents of gallic acid using the regression equation between gallic acid standards and the sample absorbance at *λ*=725 nm. The mixture is prepared by adding 0.250 mL of sample solution, 0.500 mL of Na_2_CO_3_, 4 mL of deionized water and finally 0.250 mL of the *Folin‐Ciocalteu* reagent solution (previously prepared by diluting the reagent 1:1 with deionized water). The spectrophotometric scan was performed after 25 minutes, storing the samples at room temperature in the dark.

To evaluate the phenolic content of lignin, the TPC method was slightly adapted, dissolving the sample in DMSO (the *Folin‐Ciocalteu* reagent was diluted 1:1 with a mixture of 50 % deionized water and 50 % DMSO, as well). The protocol was then conducted as previously reported. Analyses were carried out in triplicate and reported as average value ± standard deviation (SD).

*Folin‐Ciocalteau* assay allow to express as Gallic Acid Equivalents (GAE) the TPC; TPC Selectivity is reported as (mg_GAE_/g_Extr_.), while the TPC Yield as (mg_GAE_/g_Matr_.).

**Antioxidant Activity Determination (DPPH⋅ Assay)**. The antioxidant activity was assessed using the stable free radical 2,2‐diphenyl‐1‐picrylhydrazyl (DPPH⋅).^[35]^ Using methanol/water mixtures as solvents (70 % for liquid fractions and 10 % for pectins), a known concentration of sample was prepared and used to produce six to eight sequential dilutions. The absorbance of a 50 % sample solution in methanol was subtracted from the sample (matrix effect) and a solution containing 50 % of free radical (A~0.45, *λ*=515 nm) in methanol was adopted as a reference (100 %). The samples were stored at room temperature in the dark for 20 minutes before the spectrophotometric scan at *λ*=515 nm. The inhibition % is defined as per Equation 1:

(1) $$\%I=\frac{A_{ref}-A}{A_{ref}}\cdot 100$$

The IC50 (half maximal inhibiting concentration, concentration of antioxidant compounds that leads to a 50 % reduction in DPPH⋅ absorbance) was calculated plotting the %I *vs*. concentration of the samples using a Probit regression model (Software Bobo Least Squares ver. 0.9.1.).^[36]^ The standard was Trolox, and the antioxidant activity was reported as Trolox eq. (μmol_TE_/g_Extr._). Only results with a R^2^ of 0.96 or higher were considered. Analyses were carried out in triplicate and reported as average value with the relative confidence interval (CI).

#### Antimicrobial Effect of Extracts in Vinification

**Growth inhibition**. *Brettanomyces bruxellensis* ISE 373 and *Acetobacter pasteurianus* ISE 5319 were used in these assays, they belong to the Culture microbial collection (CMVE) of CREA‐VE Asti.

Initially, pre‐cultures were prepared in YEPG (1 % peptone, 1 % yeast extract, 2 % glucose) or MRS (De Man, Rogosa and Sharpe purchased by Sharlau Italy, with the following composition: 10 g/L peptone; 8 g/L meat extract; 4 g/L Yeast extract; 20 g/L glucose; 5 g/L sodium acetate; 2 g/L Tri‐ammonium citrate; 0.2 g/L Magnesium sulphate; 0.05 g/L Manganese sulphate; 2 g/L Di‐potassium phosphate; 1 g/L Polysorbate 80) medium in a biological safety cabinet with laminar flow (STERIL‐POLARIS) using yeast or bacterial stocks stored at −80 °C. For each strain, 100 μl of stock was added to 10 ml of YEPG or MRS medium. The cultures were then incubated at 30 °C in an incubator (Sanyo CFC Free MIR − 253). After 48 hours of growth in the incubator, the cultures were used for the experiments. The experiments were carried out following the method proposed by García‐Ruiz *et al*.,^[37]^ with some modifications. Briefly, a 15 mg/mL stock solution was prepared using dried Nebbiolo L1.1 fraction (extracts after pectins precipitation).

Subsequently, culture media were prepared under a laminar flow cabinet. The extract concentrations screened were 1, 2 and 4 mg/mL.

The experiments were conducted in sterile 96‐well plates (Starlab CytoOne); each selected strain was inoculated in triplicate. Strains cultured without extracts were taken as positive control. Culture media without inoculum and extracts were used as the negative controls.

Microbial growth was determined by measuring the optical density at 600 nm (OD_600_) using a microplate reader (BioTek, 800 TS) equipped with BioTek Gen6 software. The instrument was set to automatically read the OD_600_ of each well every two hours for 72 hours after 10 seconds of shaking.

**Application of Extracts in Wine**. Moscato must was subjected to fermentation and inoculated with the commercial yeast starter JB3 (Oenobrands, France), previously rehydrated in a 5 % sucrose solution following the manufacturer′s instructions. (NH_4_)_2_HPO_4_ was added to the must to ensure nitrogen nutrition for the yeast during alcoholic fermentation. The fermentation progress was monitored by tracking the weight loss. On the initial must, Brix, volatile acidity and pH were quantified, along with inorganic nitrogen and organic nitrogen. The wine obtained at the end of fermentation was tested for alcohol content, total acidity according to the OIV methods. Acetaldehyde and total SO_2_ were quantified with CDRWineLab analyzer.

The wine obtained was then divided into four aliquots for the preparation of different samples:

– No additives: the wine was bottled without any addition;
– 200 mg/mL SO_2_: 200 mg/L of SO_2_, corresponding to the legal limit was added to the wine, was added to the bottle;
– 100 mg/mL SO_2_+5 mg/mL L1.1: addition of polyphenolic extract (L1.1) and half of the total SO_2_ were added to the bottle;
– 5 mg/L L1.1: addition of sole L1.1 (5 g/L).

The wine was then put in 100 mL bottles, closed with a crown cap and conserved at 15 °C. After 3 months of refining, acetic acid, total polyphenol index (TPI), TPC (*Folin‐Ciocalteu* Assay), tannins, catechins, acetaldehyde, and color were quantified using CDR WineLab analyzer. All these analyses were conducted in duplicate.

The four samples were plated on WL agar to detect the colony‐forming unit (CFU) count and evaluate the effectiveness of each treatment in inhibiting microbial growth.

### Recycling and Reduction of Solvents and Additives

**Membrane Treatment of L1**. The extract (L1) was subjected to nanofiltration (NF) using a laboratory‐scale membrane filtration skid (PB100, Hydro Air Research Srl, Lodi, Italy). The system is composed of a 3 L tank equipped with a DKU 1812 membrane (150 – 300 Da, 0.38 m^2^ filtering area). The retentate was continuously recirculated in the feeding tank, while the permeate was collected in a graduated cylinder, which was also used to monitor the filtration flow. The solution (1.8 L, obtained from the extraction of 120 g of GS) was processed using Transmembrane Pressure (TMP, 10 bar). An aliquot (10 mL) of retentate was freeze‐dried, while the rest was subjected to spray‐drying, a less energy‐consuming technique with greater industrial scalability. The rejection index of polyphenols was calculated using Equation 2:
(2)

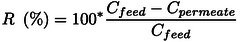




**L1 Spray‐Drying**. The spray‐drying process was selected as an alternative method for the removal of water. The system (KD‐SD‐2000, Zhengzhou Keda Machinery and Instrument Equipment Co., Ltd., Zhengzhou, China) is equipped with a peristaltic pump, an atomizer, an air heating system, a drying chamber, and a cyclone separator. A total of 490 mL of permeate was processed using an atomizer temperature of 180 °C, a venting percentage of 70 %, and a peristaltic pump speed of 30 %. The tests were conducted without the addition of any excipients, as it was anticipated that pectin would serve as a suitable substitute for maltodextrins as a support. The TPC was quantified following spray‐drying to ascertain that no degradation of polyphenolic compounds had occurred during the process.

**Membrane Treatment of L2**. L2 was subjected to NF‐DF (nanofiltration‐diafiltration) process, to concentrate the liquor and recover NaOH in the permeate, thus reducing the overall consumption of additives for the precipitation of pectin and lignin (H_2_SO_2_ and EtOH). The process was conducted on the same membrane system as in Section “Membrane Treatment of L1”, with a TMP of 5.0 bar to minimize fouling. As concerns the NF step, the volume of L2 was reduced by 72.22 %, and the pH of the permeate was monitored each drawdown of ~500 mL. After NF, 1 L of deionized water was added to the feed. The removal of 100 mL of permeate was followed by the addition of 100 mL of water in the feed, thus maintaining the total volume constant, and operating in continuous diafiltration. This procedure was carried out until a total of 2 liters were added. Subsequently, the retentate volume was reduced down to the original 500 mL and then an additional 290 mL were taken as a permeate (retentate final volume of 0.21 L). The pH of the permeate was monitored every ~100 mL drawn. The pH of the retentate was measured at several stages: post‐NF, after the removal of the first and second liters of diafiltration, and at the conclusion of the process. The permeates of the first NF, the DF and second NF were kept separate. The NaOH concentration in each sample was calculated by pH determination. The retentate was then subjected to the same procedure described in Section” Pectin/Lignin Isolation and Characterization” to separate pectin and lignin, with the exception that instead of NaOH, 210 mL of the permeate derived from the abovementioned NF were used to neutralize the pH.

### Solid Fractions Characterization

The composition of the starting biomass and of the solid fractions recovered within the cascade process, in terms of cellulose, hemicellulose and lignin content was determined by applying a protocol based on a Natural Renewable Energy Laboratory (NREL).^[38]^

The moisture content was determined by heating the samples at 100 °C overnight in a furnace (Muffle Furnace Nabertherm GmbH, Lilienthal). The determination of the inorganic material present in the samples was carried out by calcination at 650 °C for 4.45 hours (45 minutes for the temperature ramp and 4 hours for the combustion itself). Following the two thermal treatments, the samples were cooled in a drier containing phosphoric anhydride and then weighed at room temperature. The measurements were carried out in duplicate and reported as average value.

ATR‐FTIR analyses were performed by using a spectrometer Spectrum Two UATR, Perkin Elmer spectrometer.

The thermal stability of lignin was studied by thermogravimetric analysis carried out on Perkin Elmer Thermogravimetric Analyzer TGA 4000. Approximately 20 mg of lignin were heated from 30 to 900 °C, raising the temperature at a rate of 10 °C/min. A continuous nitrogen flow rate of 30 mL/min was maintained.

### Fermentation

**Inoculum**. Cellulosic substrates were fermented by *Clostridium thermocellum* LL1111 or LL1630.^[39]^ *C. thermocellum* LL1111 is a lactic acid overproducing strain obtained through deletion of its bifunctional aldehyde/alcohol dehydrogenase AdhE and expressing a mutant lactate dehydrogenase (Ldh^S161R^) which is independent from fructose‐1,6‐bisphosphate allosteric activation. The LL1630 mixed culture was obtained by *in vivo* laboratory evolution of strain LL1111 and shows a lactic acid‐hypertolerant (up to 35 g/L) phenotype. Cells were grown in anaerobic (855‐Series Anaerobic Chamber, Plas Labs Inc.) MTC‐5 chemically defined medium at pH 7.3 (Table S1).^[40]^

The carbon source of the inoculum media was 5 g/L cellobiose. The strains LL1111 and LL1630 were inoculated into separate mediums and allowed to grow until reaching an OD_600_ = 0.6.

**Tube/Flask Fermentation Without pH Regulation**. Commercial crystalline cellulose (Avicel®, Merck‐Sigma Aldrich) or the cellulose‐rich solid product (S2) obtained from the cascade process were used as the carbon source for *C. thermocellum*. The chosen final concentration of the carbon source was 50 g/L. Since Avicel® and S2 are insoluble, the sterilization was carried out by autoclaving the solution A directly in the flask and adding the other solutions under the sterile hood.

The flasks were left overnight in the anaerobic chamber and inoculated with either 0.5 mL or 5 mL of cellobiose‐grown inoculum for 50 mL flasks and 5 mL tubes, respectively. The fermentation was carried out in a glovebox incubator at 55 °C without shaking. The experiment was conducted in triplicate.

For flasks fermentation, the pH of the fermentation medium was monitored every 24 hours to observe the pH decrease during this period and confirm the production of acids resulting from *C. thermocellum* metabolism. Media samples were collected at t_o_ and t_f_ for subsequent HPLC analysis. The fermentation was interrupted when the pH stopped lowering.

**Bottle Fermentation with pH Regulation**. Using either Avicel® or S2 as the substrates, fermentations were carried out in 0.5 L anaerobic bottles (100 mL of growth medium). The medium was made anaerobic by N_2_ gas purging. The bottles were inoculated with a 5 % volume of inoculum, then incubated at 55 °C and shaken at 140 rpm. The pH was kept in a range between 6 and 8 by manual supplementation of sterile anaerobic 8 N KOH. The fermentations were carried out in triplicates and stopped after 96 hours.

**Fermentation Product Analysis**. The products formed during fermentation were identified and quantified by HPLC (Agilent Technologies 1260 HPLC), using an Aminex HPX‐87H (BIORAD) column, a flow rate of 0.6 mL/min and 0.25 g/L H_2_SO_4_ as the eluent. The samples (400 μL) were acidified by the addition of 10 % H_2_SO_4_ (20 μL) and were then vortexed, filtered and injected into the HPLC.^[41]^ The results were compared to standard products of the *C. thermocellum* metabolism, including cellobiose, glucose, acetate, lactate, formate, pyruvate, succinate, malate and ethanol. The detectors used were a UV/Vis detector (Multi Wave Detector: MWD, Agilent Series 1260) set at 210 nm and a Refractive Index Detector (RID, Agilent Series) set at 35 °C.

The significance of the difference in the final fermentation profile between strains LL1630 and LL1111 was verified trough heteroscedastic, two tails t‐student test.

**Consumed Cellulose Quantification – Phenol‐Sulfuric Acid Method**. This assay was used to measure cellulose consumption, hence, to calculate product yield. The yield was calculated using Equation 3:

(3) $$Yield=\frac{g\;ofproduct}{g\;ofcellulose\;consumed-g\;of\;sugars\;in\;solution}\;\;$$

Cellulose quantification was determined by the phenol‐sulfuric acid method as described by Updegraff (1969) and Dubois *et al*. (1956) with some modifications.[^42^, ^43^] Biomass (0.25 g) was washed with 10 mL water (4500 rpm, 15 min) (this step was skipped for S2, since it had already been washed and lyophilized). Subsequently, the biomass was resuspended in 3 mL of acetic‐nitric reagent using a vortex, incubated for 30 min at 100 °C and centrifuged (4500 rpm, 15 min) to remove lignin and hemicellulose (in the supernatant). The pellet was then washed twice with 10 mL water, re‐suspended in 67 % (v/v) H_2_SO_4_ and incubated for one hour under stirring at room temperature to promote cellulose hydrolysis. Total carbohydrate quantification was performed by the phenol‐sulfuric acid method Dubois *et al*. (1956), using glucose for the standard curve. This test was conducted in duplicate for all samples.

## Results and Discussion

### Cascade Protocol

The aim of this manuscript is to design a zero‐waste valorization protocol for grape stalks, able to efficiently exploit the complex composition of the matrix and obtain added value products from all the different components. according to the Green Chemistry and Circular Economy principles. For this reason, the approach is structured with three sequential steps: i) extraction; ii) delignification; iii) fermentation (see Figure 1). The first step of MASWE allowed the extraction of pectin and high added‐value, bioactive molecules (Figure 1, L1.1), further studied as wine preservatives. The extraction was performed at the beginning of the entire protocol, since these compounds are potentially prone to degradation during the next stages. The solid residue remaining after extraction (Figure 1, S1), besides containing still some pectin, was mainly composed of the structural components, such as cellulose, hemicellulose and lignin. The latter was removed in a subsequent step prior to the fermentation. Indeed, for the further fermentation step, a delignification treatment is required, enriching the relative percentage of fermentable material in the solid substrate (Figure 1, S2) and reducing at the same time the inhibition effects due to lignin‐derived phenolic components. The alkaline treatment used for the delignification step allowed the recovery of pectin and lignin in the liquid fraction (Figure 1, L2), and, after pectin precipitation, the supernatant (Figure 1, L2.1) was further treated with acid to pH=2 to precipitate acid insoluble lignin and recover lignin‐derived phenolic compounds in the liquid fraction (Figure 1, L2.2). The last step of fermentation has the crucial task to selectively produce LA with engineered strains of cellulolytic bacteria acting on a cellulose‐rich substrate (Figure 1, S2). According to the cascade protocol, it is possible to draw the process scheme dividing the production streams into liquid and solid fractions (Figure 1), helping to simplify and summarize all the results (see Section “Liquid Fractions” and Section “Solid Fractions”). The detailed scheme, completed with products weights and yields, is reported in Figure S1. According to this approach, the first tests on GS were carried out to design a suitable extraction protocol and, in particular, to define a convenient cultivar of grapes that could be exploited as a reference matrix for the whole process.

### Benchmark Extraction

A conventional extraction was carried out to quantify the total extractable polyphenols present in different cultivars from Piedmont region, such as Barbera, Dolcetto and Nebbiolo. As reported in Table 1, Dolcetto results in a slightly better TPC yield compared to Barbera, but Nebbiolo is the best sample in term of phenolic content, compared to the other two cultivars.

**Table 1:** TPC Yield comparison between cultivars.

Cultivar	TPC Yield (mg_GAE_/g_Matr._)	S.D.	TPC Selectivity (mg_GAE_/g_Extr._)	S.D.
Barbera	104.77	± 2.36	229.09	± 2.88
Dolcetto	124.54	± 1.49	274.62	± 2.06
Nebbiolo	198.37	± 2.61	370.55	± 1.99

### Extraction Screening (MASWE)

**Figure 2:**
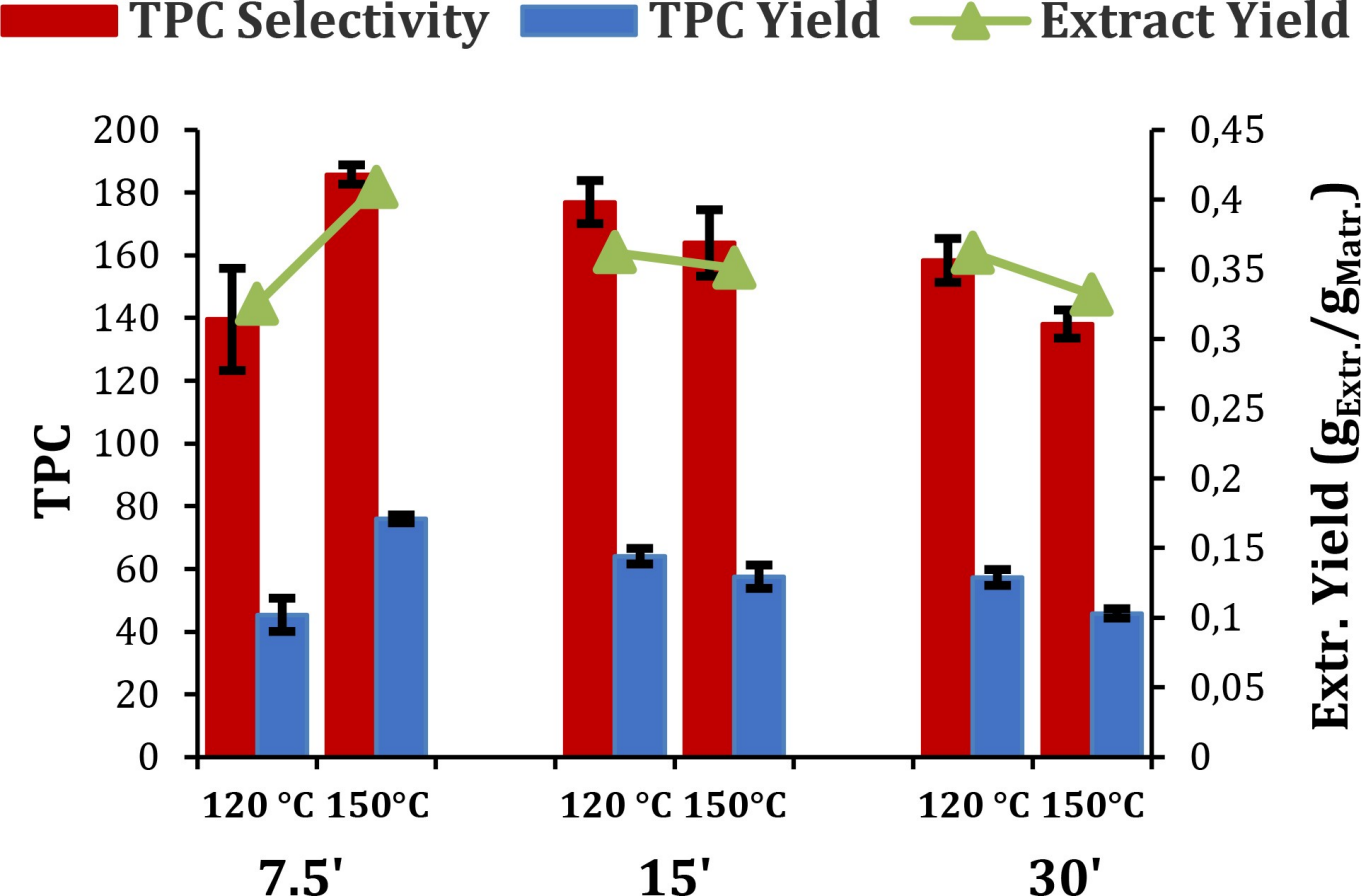
MASWE screening of Nebbiolo cultivar, reported in terms of TPC (Yield expressed as mgGAE/gMatr and Selectivity expressed as mgGAE/gExtr) and extract yield.

To evaluate the efficacy of MASWE, temperatures of 150 and 120 °C were investigated along with extraction times of 7.5, 15 and 30 minutes. Since Nebbiolo had the highest polyphenols content, this cultivar was selected as reference matrix (Figure 2).

The best results were achieved at 150 °C after 7.5 min, affording 185.78 mg_GAE_ / g_Extr_ and 75.99 mg_GAE_ / g_Matr_, in terms of TPC selectivity and yield, respectively. Considering the trend reported in Figure 2, it can be generally deduced that higher temperatures are efficient for short treatments, since prolonged ones result in a general drop in TPC values, as well as extraction yields. This can be due to the thermal degradation of the polyphenols, which occurred when harsher conditions are applied for longer times.^[44]^

**Figure 3:**
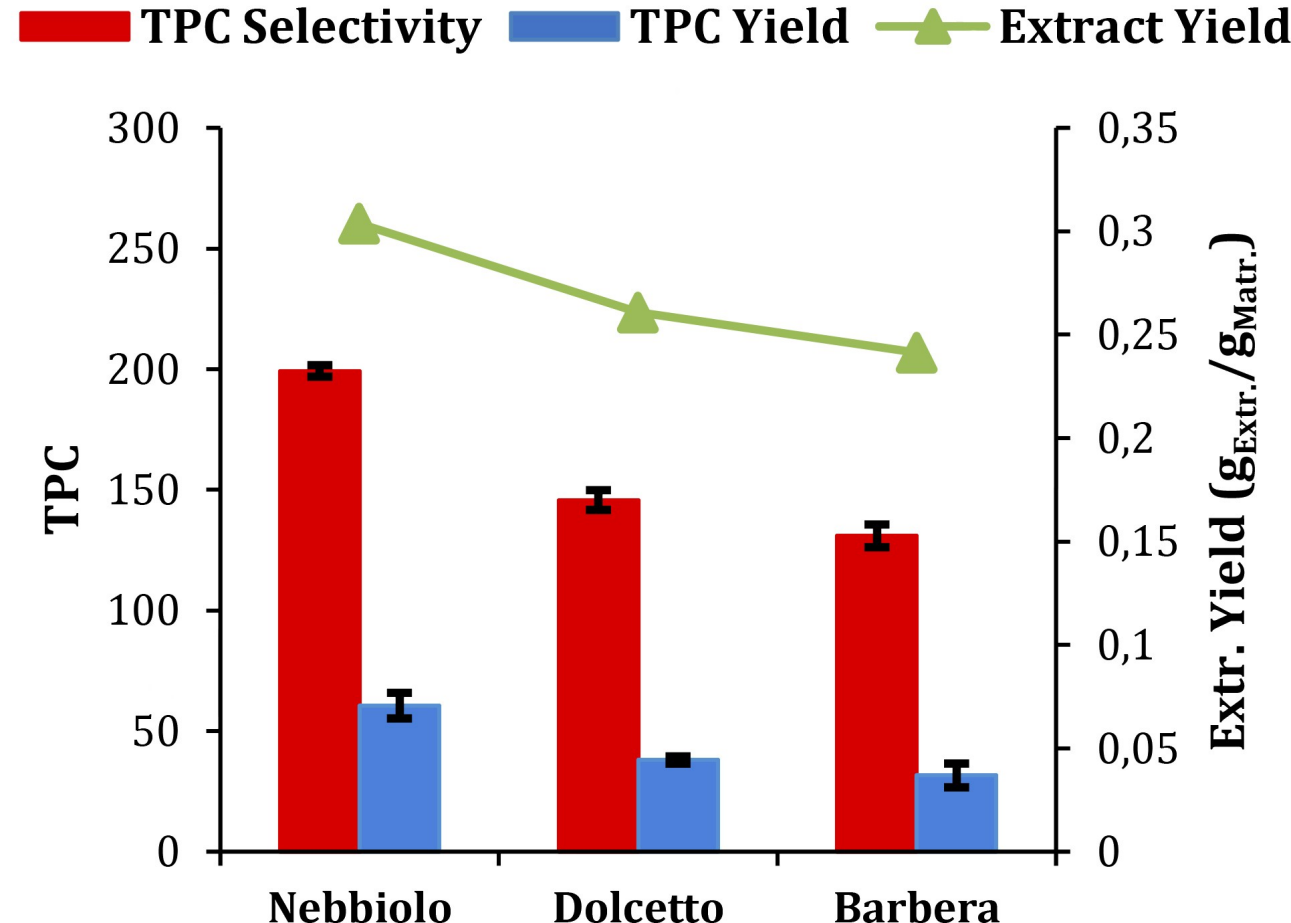
MASWE screening with optimized conditions on different *cultivars; TPC* Yield expressed as mg_GAE_/g_Matr._, TPC Selectivity expressed as mg_GAE_/g_Extr_.

The optimized extraction conditions were then applied for the treatment of Dolcetto and Barbera cultivars (Figure 3).

A certain heterogeneity in chemical profiles is worthy of note among different cultivars. However, the results confirm that Nebbiolo is the most promising cultivar, for the further valorization of polyphenols. Hence, it was selected for the cascade valorization process. The results and discussion about this protocol will be separately reported for liquid and solid production streams (Figure 1).

### Liquid Fractions

**Figure 4:**
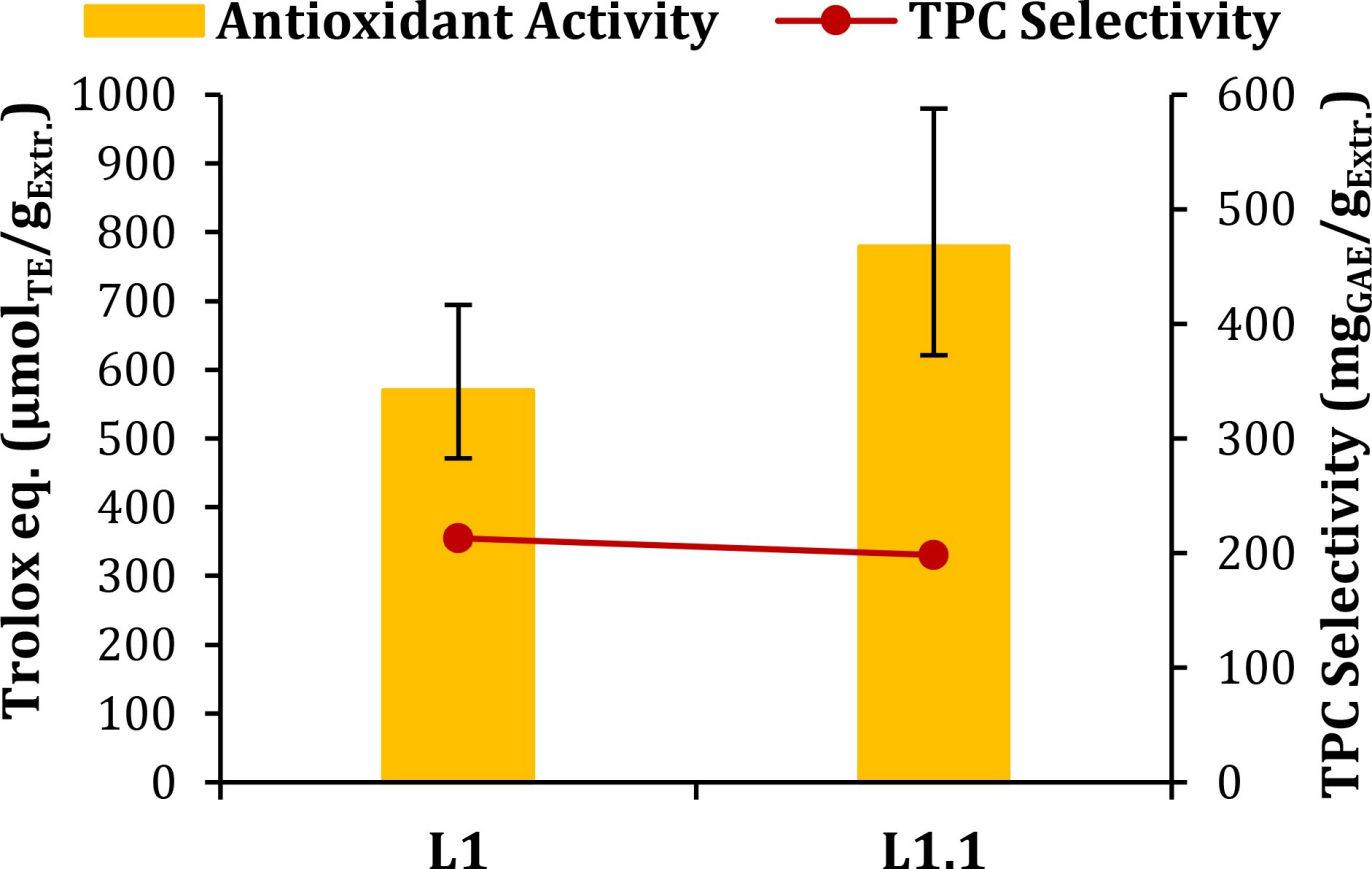
TPC selectivity and Antioxidant activity of L1 and L1.1.

**Extraction Step**. The first extraction step of the cascade process afforded a raw liquid fraction (L1, Figure 1). Thanks to a simple anti‐solvent approach (see Section “Pectin/Lignin Isolation and Characterization”), it is possible to precipitate the pectin contained in L1, thus affording a liquid fraction enriched in polyphenols (L1.1, Figure 1). As shown in Figure 4, the pectin removal led to a slight decrease in TPC selectivity (from 213.0 to 198.36 mg_GAE_ / g_Extr_). This result suggests a possible loss of polyphenols during the precipitation with anti‐solvent due to their conjugation with the polysaccharides.^[45]^ The presence of phenolic compounds in the precipitated pectin fraction was also confirmed by ATR analysis (see Section “Pectin”).

Conversely, considering the antioxidant activity, an increase of 36.6 % is recorded for L1.1 (779.29 μmol_TE_ / g_Extr._, compared to 570.35 μmol_TE_ / g_Extr._ for L1), probably due to an increased concentration of antioxidant polyphenols.

**Figure 5:**
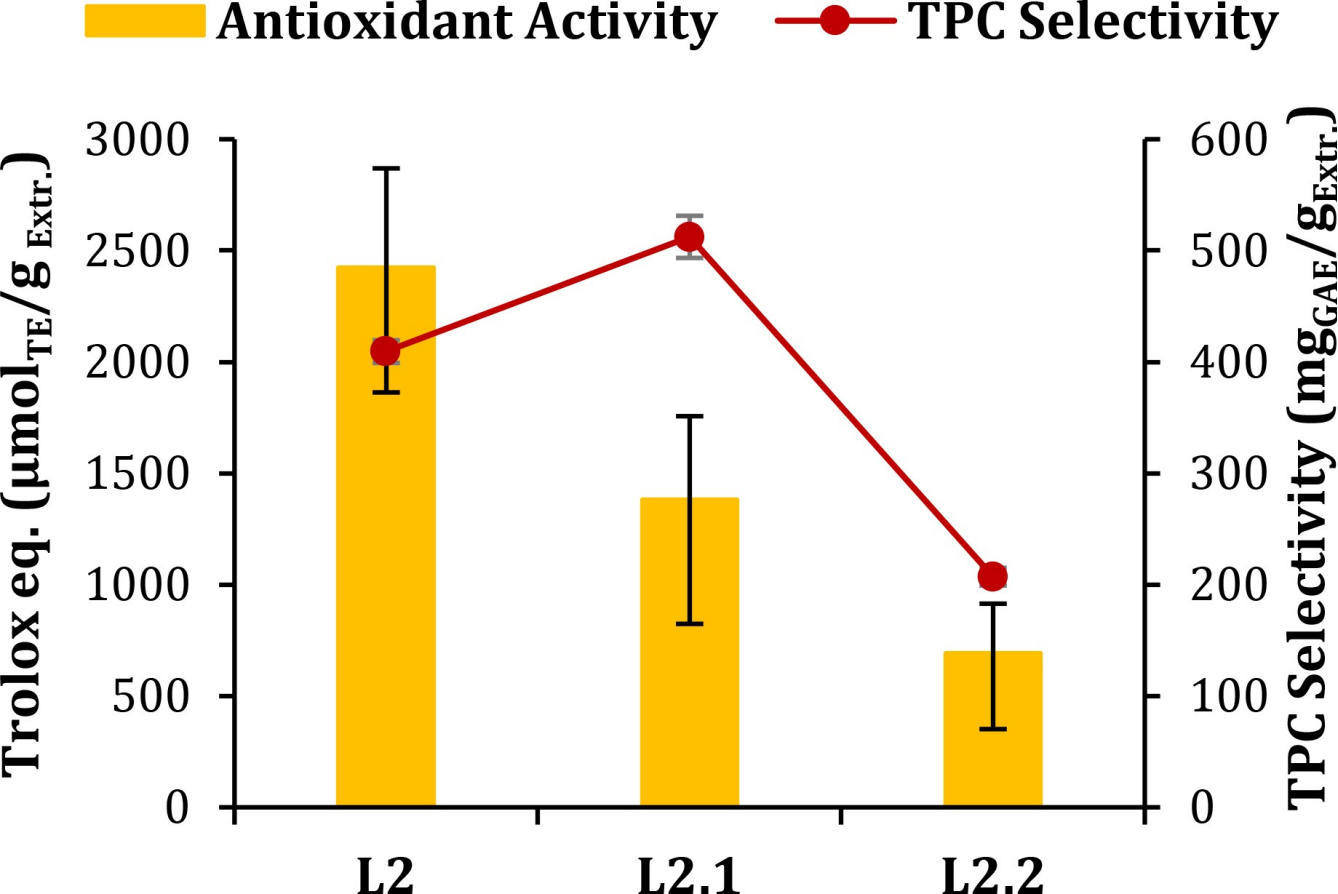
TPC Selectivity and Antioxidant activity of L2, L2.1 and L2.2

**Delignification Step**. As mentioned in Section “Cascade Protocol” 3.1, the post‐extraction solid residue (S1) underwent a MW‐assisted delignification step, affording a liquor (L2, Figure 1) that can be furtherly fractionated by the sequential precipitation of pectin (L2.1, Figure 1) and acid‐insoluble lignin (L2.2, Figure 1), leading to a liquid fraction (L2.2) containing acid‐soluble lignin. As shown in Figure 5, delignification produces a liquid fraction with a high content of polyphenols per gram of dry extract, two‐fold higher than L1 (409.24 mg_GAE_ / g_Extr._ *vs*. 213.00 mg_GAE_ / g_Extr._). This can be due to the phenolic nature of extracted alkali lignin. The precipitation of pectin causes a noticeable increase of TPC selectivity (*approx*. +25 %) in L2.1. On the other hand, this parameter dramatically drops after the precipitation of acid‐insoluble lignin. This data supports the hypothesis that lignin moieties are the main actors in the TPC output. However, a certain phenolic content still is observed in L2.2 (207.13 mg_GAE_ / g_Extr_), thus indicating the permanence of small acid soluble molecules resulting from the depolymerization under alkaline conditions.^[46]^

Taking into consideration the antioxidant activity, it is possible to notice a constant drop in Trolox eq. from L2 to L2.1 and L2.2 (2421.81, 1380.85 and 690.43 μmol_TE_/g_Extr_, respectively). Most likely indicating the precipitation of antioxidant substances. The darker color of the polysaccharidic fractions precipitated from L2, compared to the one recovered after extraction, supports the hypothesis of the different nature of metabolites trapped inside the galacturonic cage (Figure S2). According to literature, the co‐precipitates could be mainly composed by proanthocyanidins, as their strong affinity to pectin has already been demonstrated, since positively charged cyanidin‐3‐glucoside has been found to bind the negatively charged pectin‐containing cell wall through a charge‐attraction mechanism.^[47]^ Moreover, the pectic fraction of *Vitis vinifera* cell wall has been found to strongly bind proanthocyanidins.^[48]^ This class of polyphenols were solubilized in the delignification step rather than during MASWE as they are usually efficiently extracted in alkaline conditions, being normally tightly bound to lignin and cellulose structures,^[49]^ which are respectively disrupted and altered during the delignification step. These findings could be ascribed to the chemical difference between both L1‐derived pectin, L2‐derived pectin and the polyphenols solubilized in the two liquid fractions. Indeed, it has already been observed that the capacity of pectin to incorporate polyphenols depends on their porosity, on the presence of hydrophobic cavities and on the presence in their structure of Ca^2+^ ions, which have been found to be capable of complexing antioxidant substances within the polymer.^[50]^ Furthermore, it has been shown that different polyphenols have different affinities towards pectin.^[51]^

**Antimicrobial Effect of Polyphenolic Extracts**. As is well known the antimicrobial effect of polyphenols, we decided to test the possible application of these extracts against the species that could affect wine production, according to a circular economy approach.^[52]^

Two different species were tested to assess the antimicrobial effect: the bacterium *Acetobacter pasteurianus* and the yeast *Brettanomyces bruxellensis*. These microbes can drastically alter wine quality. Acetic Acid Bacteria such as *Acetobacter*, can multiply in wine and produce chemicals like acetic acid (vinegar), acetaldehyde (pungent smell), and ethyl acetate (solvent‐like aroma). These alter the taste and smell of the wine, making it unenjoyable.^[53]^ This bacterial growth can happen at various stages of winemaking.

*Brettanomyces*/*Dekkera* can grow during winemaking, it produces unpleasant aromas described as “phenolic” (band‐aid like), “animal”, and “stable”. These unpleasant odors come from ethylphenols and related compounds produced by this yeast.^[54]^ These characteristics make *B. bruxellensis* a major concern for winemakers and cellar managers.^[55]^

**Figure 6:**
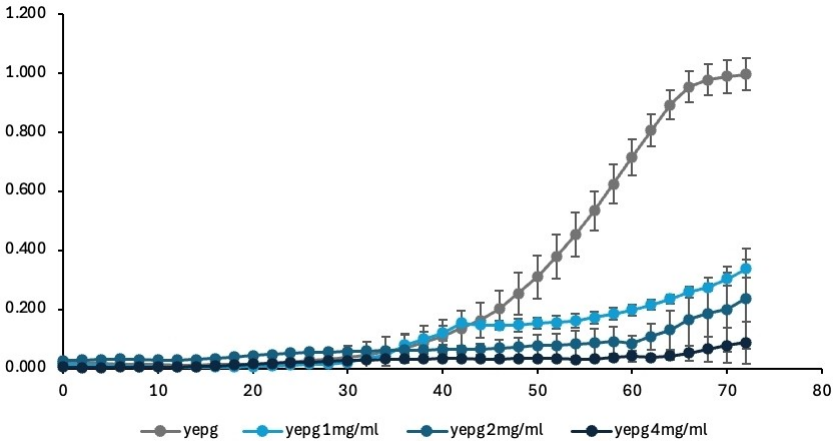
Growth of *B. bruxellensis* measured by absorbance (OD_600_) over time in hours; the control without extracts is indicated as YEPG, the other samples were in YEPG added with 1, 2 and 4 mg/L of L1.1

Figure 6 shows the effect of L1.1 on the growth of *B. bruxellensis*. It can be observed that in the presence of polyphenols the growth of this yeast is delayed with all the tested polyphenol amounts, with the highest inhibition achieved at 4 mg/mL of extract.

**Figure 7:**
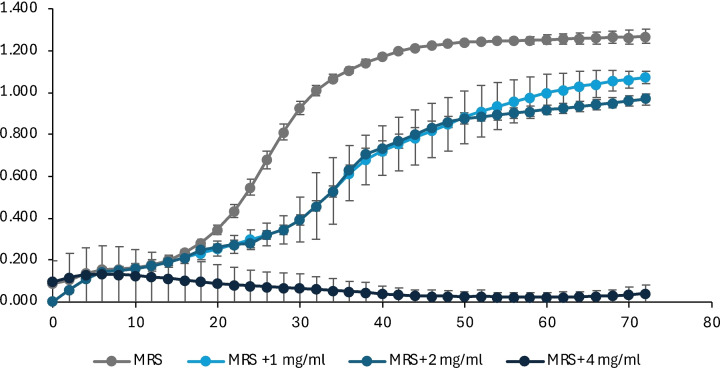
Growth of *A. pasteurianus* measured by absorbance (OD_600_) over time in hours; the control without extracts is indicated as MRS, the other samples were MRS added with 1, 2 and 4 mg/L of L1.1.

In Figure 7 the effect of the tested compounds on *Acetobacter* growth is shown. In this case with 1 and 2 mg/L the exponential phase is slightly delayed but at the end, after 72 hr, the final optical density is similar to the culture without the extracts. While 4 mg/L of polyphenols in the medium blocked the *Acetobacter* growth.

In general, it can be stated that the effect of polyphenols varies according to the different species. A good effect was observed in *Brettanomyces bruxellensis* while in *Acetobacter pasteurianus* a significative result was detected only applying a higher dose of polyphenols. García‐Ruiz *et al*.^[56]^ tested different phenolic compounds on *Pediococcus pentosaceus* and *Lactobacillus hilgardii*, two lactic acid bacteria found in wine, and showed that the minimum inhibitory concentrations varied according to the type of compound: for example, gallic acid had a MIC was 300 mg/L for *Lactobacillus* and 200 mg/L for *Pediococcus*. Thus, they used higher amounts of polyphenols than we do.

Generally, in this study, a greater effect can be noticed on yeasts compared to bacteria. Recently Gutiérrez‐Escobar *et al*.^[57]^ extracted stilbenes from grapevine shoots and assessed its antimicrobial properties, they observed a good antimicrobial activity which was more efficient for yeast than for bacteria in wine, which agrees with our observations.

This matter should be further investigated: it is necessary to study different strains of the same species to assess if the effect of polyphenols is the same or if it is strain‐dependent, as it could pave the way for a more sustainable control of contaminant species during vinification, according to a circular approach.

**Application of Polyphenolic Extracts to Vinification**. Moscato wine was fermented using the commercial starter JB3, the parameters of both must and wine are reported in Table S2 and Table S3, respectively.

The color of the wine is significantly affected by the addition of extracts, as shown in Table S4 and, more precisely, in the Post Hoc Tukey test displayed Table S5E.

Volatile acidity increased in all tests compared to the initial wine, but it was particularly high when only extracts were added. These results are not clear, since the increase of volatile acidity can be due to oxidation and the presence of extract should prevent this phenomenon due to their antioxidant properties.

The Turkey test revealed that the samples treated with SO_2_ and the one treated with both SO_2_ and L1.1 belong to the same group (Table S5A), hence the addition of polyphenols reduces the SO_2_ concentration required to prevent the increase in volatile acids.

Samples where L1.1 was added had significantly higher levels of catechins, Abs 420, IPT, and polyphenols compared to other treatments (Table S5B, C, D, E). In particular, the combination of L1.1 and SO_2_was found to effectively fortify and protect the quantity of polyphenols in wine.

The samples were also plated on WL agar to evaluate microbial growth: in the samples treated with SO_2_ no growth was observed, while in both untreated sample and sample treated with only L1.1, a growth of 4000 CFU/mL was detected.

Therefore, it could be concluded that extracts alone do not possess antimicrobial activity high enough to protect wine from contamination, but their addition can help in decreasing the quantity of SO_2_ necessary to mitigate the growth of contaminants. However, the addition of extracts causes an unwanted change in color. In fact, the addition of L1.1 to white wine causes a complete change in color, from pale yellow to brown. This was probably due to the presence of antioxidant strongly colored phenolic compounds, like anthocyanins, recovered in the first extraction step.

Wine contains various phenolic compounds which, together with the aroma molecules of wine, are the main factors responsible for grape and wine quality, determining important organoleptic properties such as wine color, taste and mouthfeel.^[58]^ The wine darkening caused by the addition of L1.1 is not acceptable to the consumer, since the color change of wines is a very important qualitative parameter^[59]^ which deserves the same attention in the control of all the other parameters. For this reason, further studies should be done to optimize their application.

**Reduction, Recycling and Reuse of Solvents and Additives**. The NF of L1 (Figure S3) allowed to concentrate the volume of the extract solution by 72.22 %. The process lasted 13.6 minutes, during which the membrane exhibited 26.27 L/(h ⋅ m^2^) as a top flow performance. Starting from a feed with a concentration of 21 g/L, the NF protocol produced a retentate and a permeate with a concentration of 64.78 g/L and 2.46 g/L, respectively. In particular, the substantial reduction in the concentration of permeate makes it suitable for its recycling within the process, for example as solvent for further cycles of extraction or for the diafiltration process. On the other hand, the rise in retentate concentration affords an increase in polyphenols concentration of 312 % (corresponding to a rejection index of 95 %, for total polyphenols). Concurrently, the high concentration makes the retentate an ideal candidate for spray‐drying, a technique proven to be faster and more energy‐effective if compared to freeze‐drying, without affecting TPC stability. On the contrary, the pectin inherently present acts as a stabilizer, encapsulating the polyphenols in the extract, thus improving the process.[^60^, ^61^]

An alternative approach entails the removal of pectin from the retentate, thereby affording L1.1, which requires 72.22 % less ethanol compared to the downstream process carried out in the absence of the membrane treatment (Figure S3).

In the following delignification step, the pH of the liquid phase decreased from 12.88 to 9.91, as NaOH reacted with the acidic groups of S1, resulting in the consumption of 99.94 % of the base. This indicates that the quantity of NaOH utilized for delignification is suitable to react with the acidic groups of lignin in almost stoichiometric amount, thus minimizing the waste generation. The NF‐DF process (Figure S4) resulted in a further reduction of the pH from 9.91 to 8.74, indicating that 99.06 % of the remaining NaOH after the reaction with lignin was removed from the retentate and recovered in the permeate (Table S6). Figure S5 illustrates that the highest permeate pH (consequently the highest NaOH removal), was achieved with greater dilutions of the retentate during continuous diafiltration. As the volume of the retentate decreased, the efficiency of NaOH removal also declined. This thesis has been demonstrated by discontinuous NaOH diafiltration, which resulted in a lower alkali removal (94.30 %) in relation to an extensive use of water (4 L) (Figure S6). The diafiltration treatment resulted in a reduction in NaOH and thus a lower amount of H₂SO₄ was required to neutralize L2.2. Consequently, the Na_2_SO_4_ content of L2.2 is strongly reduced (by 43.50 %), if compared to the downstream process in the absence of the membrane treatment.

**Figure 8:**
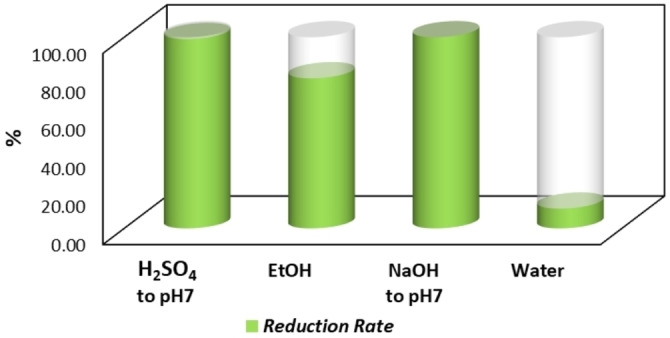
Reduction of solvents and additives after membrane downstream

As illustrated in Figure 8, NaOH diafiltration allowed a reduction of 99.06 % H_2_SO_4_ amount required for the precipitation of pectin and lignin, compared to the acid used in the protocol without membrane treatment. The alkaline NF permeate was then employed to neutralize L2.2 after lignin precipitation (Figure S4), thereby avoiding any further NaOH addition in the downstream process of L2 treatment. In comparison to the cascade process without membrane treatment, the overall concentration achieved by NF protocols of L1 and L2 afforded a global reduction of 78.53 % in ethanol employment. Finally, the reuse of the permeate from the NF of L1 can lead to a 10.35 % reduction in water consumption across the entire membrane process.

Regarding the reuse of water, it should be noted that further studies could enable the recycling of the NF‐DF permeate, resulting in a potential water saving of up to *approx*. 74 %. Another possible approach is the reverse osmosis to separate water from NaOH. The latter could be recycled for the delignification step, which would further improve the overall sustainability of the valorization process.

### Solid Fractions

**Pectin**. Pectin is known to exhibit an excellent antiradical activity, likely *via* glucoside‐bond cleavage of galacturonic acids, able to break and trap radicals.^[62]^ Hence, to assess the antioxidant features of the different isolated fractions, tests were carried out on all the precipitated pectin (see Table 2). The biopolymers recovered from the extraction step resulted as 32 % more active if compared to the source L1 (753.19 vs. 570.35 μmol_TE_/g_Extr._, respectively), with a recovery yield of 5.85 % on the processed biomass. Conversely, both samples precipitated from delignification liquor (after neutralization and the addition of ethanol) showed a lower antioxidant activity compared to L2, while it is almost the same as L1. This pectin accounts for an overall yield of 7.3 % on the processed biomass. The high antioxidant activity of Pectin L2 EtOH could be explained by the low‐esterification degree (which can be appreciated in ATR‐FTIR spectrum reported in Figure S7) that positively affect the antioxidant activity^[63]^ and increase the exposed ‐COOH groups responsible for the complexation of proanthocyanidins.^[48]^

**Table 2:** Antioxidant activity of pectin precipitated from L1 and L2.

Sample	IC50 (μg_Pect_/mL)	CI (μg_Pect_/mL)	Trolox eq. (μmol_TE_/g_Pect_)	CI (μmol_TE_/g_Pect_)
Pectin_L1_EtOH	20.9	17.2 ÷ 25.6	753.19	614.91 ÷ 915.22
Pectin_L2_pH 7	29.5	24.7 ÷ 35.3	533.62	445.94 ÷ 637.32
Pectin_L2_EtOH	12	10.1 ÷ 14.4	1311.81	1093.18 ÷ 1558.59

CI: Confidence Interval

To better characterize the pectin obtained in the cascade process, all the samples were analyzed with ATR‐FTIR. As can it be seen in Figure S8, the pectin precipitated at pH 7 from L2 presents a large band between 3000 and 3700 cm^−1^, belonging to −OH vibrational modes; furthermore, it presents a band at around 1615 cm^−1^ attributable to the stretching of non‐esterified =O groups, as well as other bands typical of pectin (approximately 1020, 1050, and 1150 cm^−1^),^[64]^ while the intense band at 1320 cm^−1^ can be assigned to the vibration of CH_2_ groups.^[65]^ To summarize, pectin precipitated at pH 7 presents a peculiar spectrum with copresence of the typical bands of pectin together with other intense bands that can indicate the occurrence of other compounds. In comparison, the ATR‐FTIR spectra of pectin precipitated in ethanol (Figure S7) shows a significant similarity and overlaps in the typical region ascribed to those biopolymers (900 and 1450 cm^−1^). As for Pectin_L2_pH7, also the spectra in Figure S8 feature a broad band between 3000 and 3700 cm^−1^, attributed to the oscillations of the −OH groups. The band around 1320 cm^−1^, assigned to the stretching of the =O bond in carboxylic groups is mainly present in the pectin precipitated from L2, where a signal at 1410 cm^−1^ is also observed, attributable to the bending of the −O−H bond. Interestingly, the spectrum of Pectin_L1_EtOH shows a band around 1730 cm^−1^, characteristic of the stretching of =O in esters. This band is not present in the pectin precipitated from L2, which instead exhibit only the one around 1600 cm^−1^, typical of the stretching of =O in carboxylic acids.[^64^, ^66^, ^67^] These findings are consistent with the fact that alkaline treatment is commonly used to demethylate pectin^[68]^ and suggest that delignification and extraction allow the obtainment of pectin with different degrees of esterification. The presence of two bands around 1323 cm^−1^ and 1409 cm^−1^ validate the hypothesis of proanthocyanidins co‐precipitation with L2 pectin, as the band around 1409 cm^−1^ is conducible to aromatic ring vibration^[69]^ and the band at 1323 cm^−1^ is caused by aromatic ring vibrations of procyanidins. Moreover, the band at 3600–3000 cm^−1^ is more pronounced, due to the presence of those condensed tannins.^[70]^ The bands at 774 cm^−1^ present in all the ATR spectra of pectin can be assigned to the −H bending of a cyclic compound with one or more substitutions, which can be attributed to polyphenolic residues. This finding supports the idea that the precipitation does not allow to recover the pure biopolymer, which is affected, in different measures, by polyphenolic co‐presence.

**Lignin**. The ATR spectrum of lignin extracted from GS (see Figure S9), is very similar to the one obtained by Salgado‐Ramos *et al*. using the same biomass and the same MW‐assisted delignification process.^[71]^ The three typical bands that correspond to aromatic skeletal vibrations (between 1420 and 1600 cm^−1^) are well emphasized.[^72^, ^73^] In the spectrum is also noticeable a shoulder around 1700 cm^−1^ which is conducible to the C=O stretching of carboxylic acids; between 2900 and 2800 cm^−1^ the bands corresponding to −CH_2_ and −CH can be found.^[73]^ The TG and DTG analysis of lignin offered a deeper insight into lignin characteristics (See Figure S10 and Figure S11). Two main weight losses were detected, the first one around 110 °C is conducible to the loss of low molecular weight volatiles^[74]^ and water, both free and deriving from dehydration reactions.^[75]^ The second weight loss is caused by the breakage of inter‐unit linkages, strictly linked to the aromatic structure of lignin and its molecular weight. The acid‐insoluble lignin obtained from the cascade process showed the maximum weight loss (T_max_) at 350 °C, which makes it a relatively highly thermostable lignin compared to others reported in literature.[^71^, ^76^, ^77^] After 480 °C, it is possible to notice a slower weight loss caused by the final decomposition of aromatic compounds, as reported in literature,[^74^, ^75^] with subsequent formation of ashes. Those latter account for 40% of the initial weight, in accordance with literature for softwood lignin (from 40 to 50%).^[78]^ A relatively high ash content has also been correlated to a higher lignin purity, since Garcìa *et al*. observed that hemicellulose contamination leads to ash contents around 20% or lower, compared to the initial sample.^[79]^ After the re‐solubilization in DMSO, the acid‐insoluble lignin was analyzed for TPC and antioxidant activity, showing a TPC of 819.18 mg_GAE_/g_Extr_, an IC50 value of 10.8 (μg_Lign._/mL)(CI: 9.1÷13) and 3107.51 μmol_TE_/g_Lign_(CI: 2581.62÷3688.03). It is worth noticing that the *Folin‐Ciocalteau* is a semi‐quantitative method, thus it is possible that condensed materials could be affected by a lower response to this test due to slower kinetics or reduced accessibility. Such high phenolic content could pave the way to further valorization of lignin, in terms of either fine or technical applications.[^79^, ^80^]

The DPPH⋅ test revealed a remarkable antioxidant activity (3107.51 μmol_TE_/g_Extr_). This result is not surprising considering the nature of the lignin framework.

**Figure 9:**
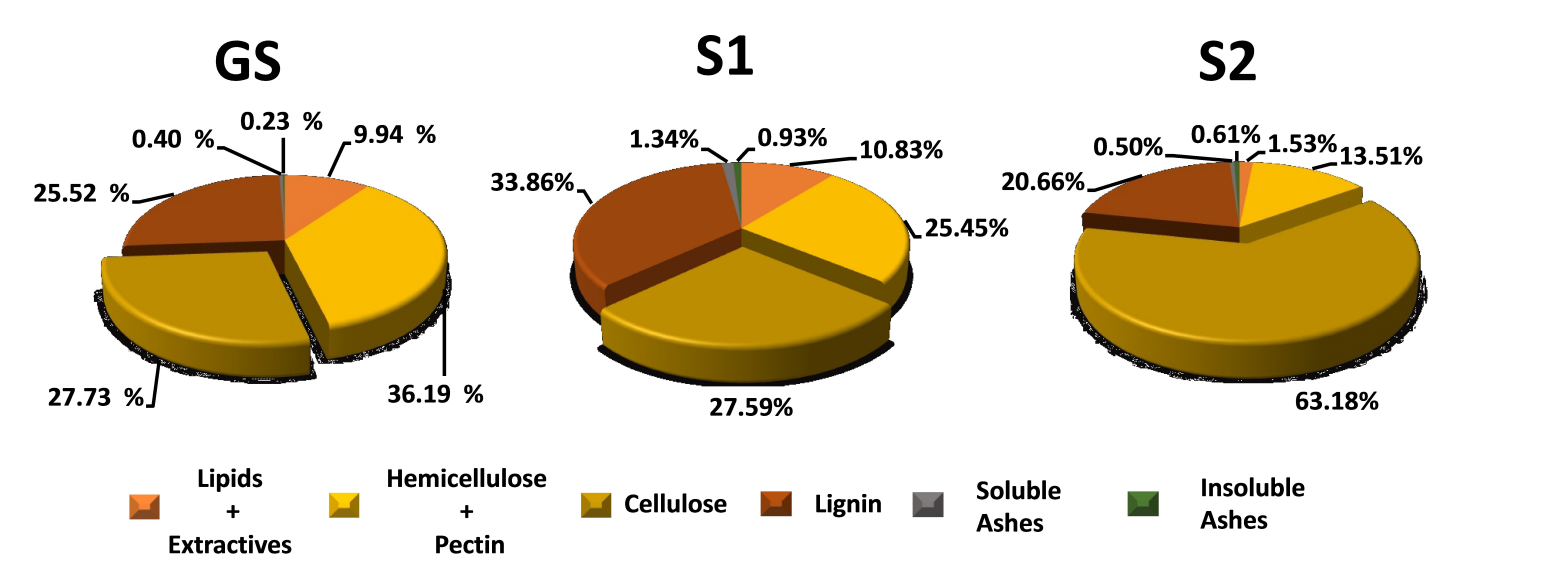
Variation of GS percent composition during cascade process

**Lignocellulosic Solids**. The cascade process produced an intermediate solid residue (S1, Figure 1), remaining after MASWE, and the final cellulose‐rich fraction (S2, Figure 1). The extraction step mainly removed extractables together with hemicellulose and pectin as primary metabolites. The delignification process caused a general depletion of all the biomass components, but with particular selectivity towards lignin and the residual hemicellulose+pectin fraction, thus resulting in a general enrichment in cellulose relative content (63.18% in S2 *vs*. 27.73% of dried stalks, Figure 9). Cellulose enrichment and purification was the main aim of the delignification step, as S2 was later used as substrate by cellulolytic bacteria for LA production.

A desirable side‐effect of the cascade treatment is the comminution of the biomass, shredding it into smaller particles thanks to the action of microwaves, heat, and continuous agitation (Figure S12). This additional pre‐treatment increased the surface area of the solid, bringing advantages for both delignification and fermentative purposes as it increases the substrate porosity and, hence, the mass transfer, due to MW irradiation.^[81]^

**S2 fraction fermentation**. S2 fraction was fermented with *C. thermocellum* strains LL1111 and LL1630, which had previously been engineered to overproduce LA.[^39^, ^82^] LL1111 is a strain in which ethanol production pathway has been eliminated; this strain is also characterised by a S161R mutation in the lactate dehydrogenase (*Ldh*) encoding gene, which makes the enzyme independent from fructose‐1,6‐bisphosphate allosteric activation.^[82]^ LL1630 was obtained through *in vivo* evolution of LL1111 and is characterized by an increased tolerance to LA (up to 35 g/L).^[39]^ Fermentations on S2 were compared to cultures supplemented with Avicel®, a commercial crystalline cellulose, that was used as the reference substrate. Recent studies have indicated that *C. thermocellum* is very sensitive to acidic pH and that its growth is severely limited at pH~6.[^39^, ^83^] For this reason, in the present investigation, fermentations were performed both with and without pH regulation.

**Figure 10:**
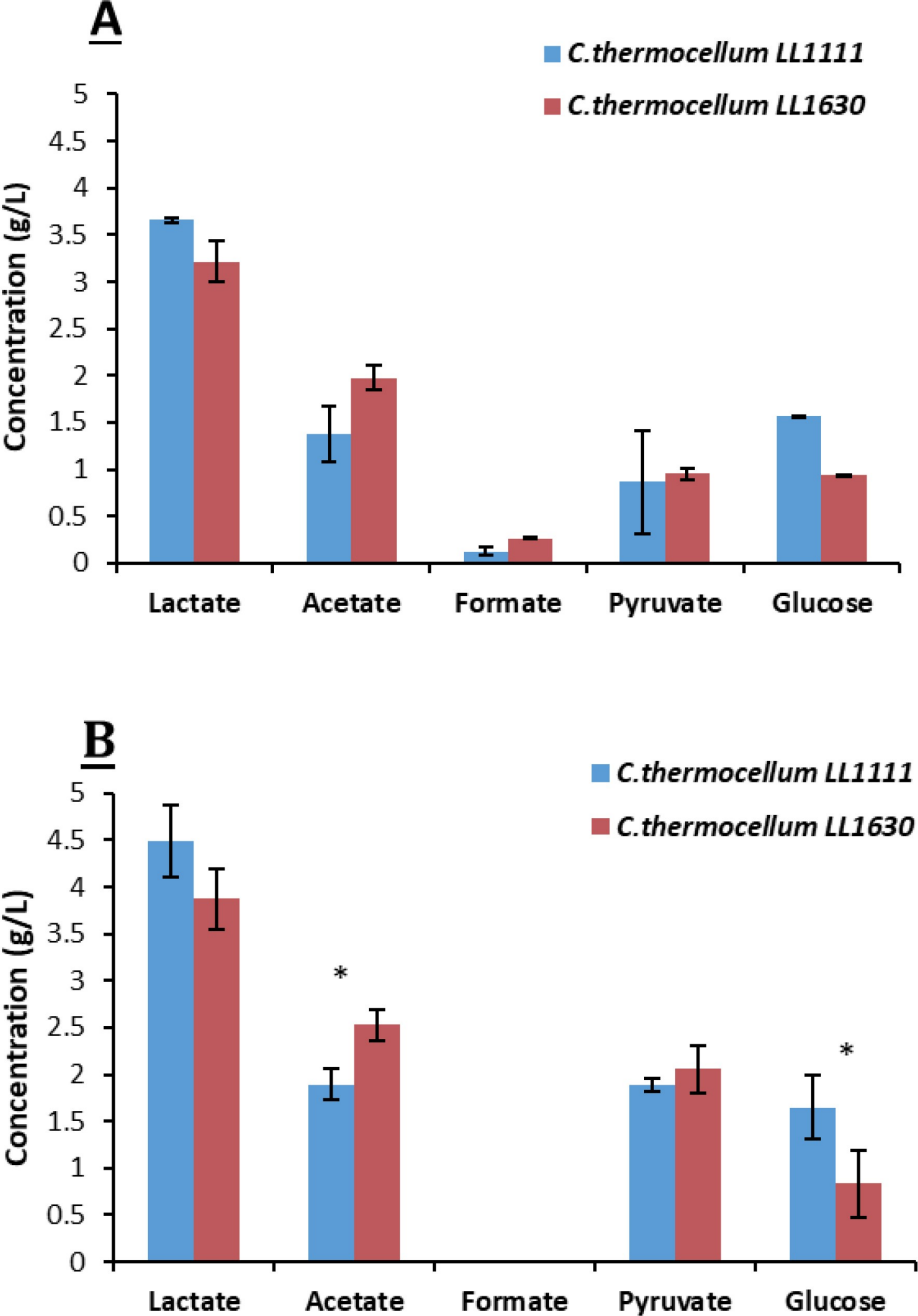
Final concentration of fermentation products with Avicel® (A) and S2 (B) (tube fermentation) (* p‐value<0.05).

**Fermentation Tests Without pH Regulation**. Tube fermentation (5 mL) assays (no pH regulation) resulted in slightly different product profile between *C. thermocellum* strains LL1111 and LL1630, but mostly not significant according to t‐test analysis (Figure 10). Irrespective of the growth substrate, LA was the main fermentation product by both strains, with a yield corresponding to 22 %–34 % of the maximum theoretical (2 mol LA/mol hexose). Interestingly, in both Avicel®‐ and S2‐supplemented cultures, substrates were only partially consumed and significant amounts of glucose (0.8–1.6 g/L) and pyruvate (0.9–2.1 g/L) were accumulated which were no further metabolized. In general, fermentation product profile did not show major differences when *C. thermocellum* strains were growing on the model substrate Avicel® or S2 fraction apart from a small amount of formate (≤0.26 g/L) which was detected in Avicel®‐grown cultures only (Figure 10). Based on these observations, S2 fraction can be considered as a suitable substrate for *C. thermocellum* fermentation.

In terms of product yield (calculated on the fraction of cellulose consumed minus the amount of glucose accumulated), no significant difference between the two *C. thermocellum* strains was detected for cultures where Avicel® was fermented (Table 3). However, strain LL1630 performed better than LL1111 on S2 (p‐value<0.05). Interestingly, strain LL1630 showed higher acetate yield than LL1111 on both cellulosic substrates (Table 3).

**Table 3:** Final product yield (tube fermentation). Yields labelled with asterisk indicate values obtained from S2‐supplemented cultures that are significantly different from Avicel*®*‐supplemented cultures (* p‐value<0.05).

C. thermocellum	Substr.	Yield (g/g)			
		Lactate	Acetate	Formate	Pyruvate
LL1111	Avicel®	0.426±0.026	0.161±0.015	0.015±0.001	0.10±0.007
	S2	0.274±0.023*	0.116±0.010	‐*	0.115±0.004
LL1630	Avicel®	0.379±0.003	0.233±0.035	0.031±0.005	0.112±0.065
	S2	0.337±0.028	0.220±0.014	‐*	0.179±0.022

**Figure 11:**
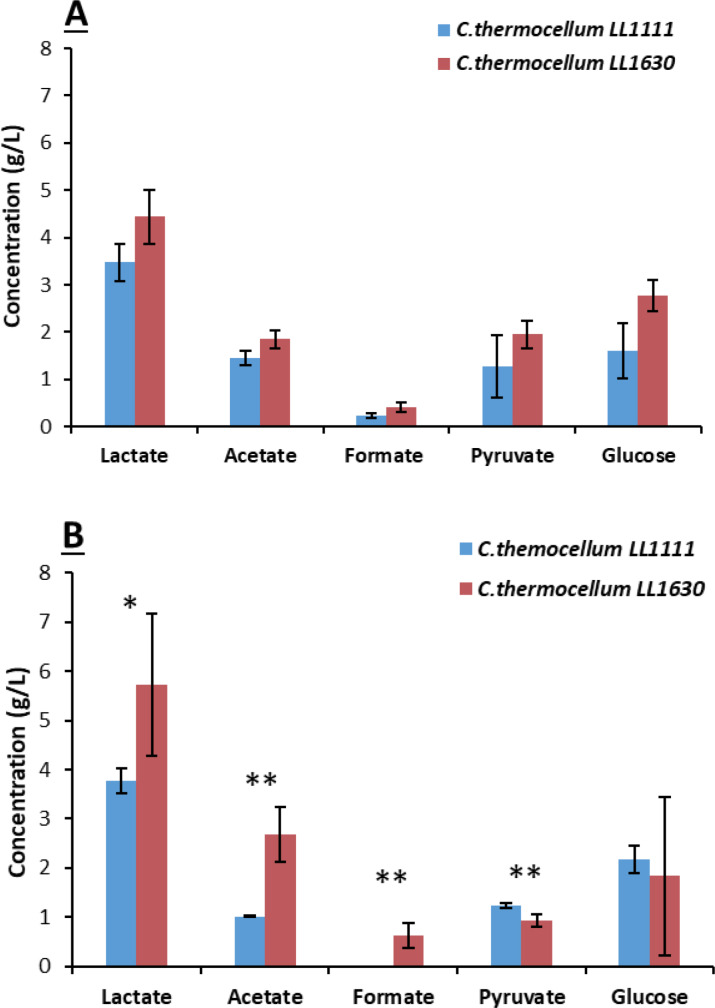
Final concentration of fermentation products with Avicel® (A) and S2 (B) (flask fermentation) (* p‐value<0.05; ** p‐value<0.01).

**Figure 12:**
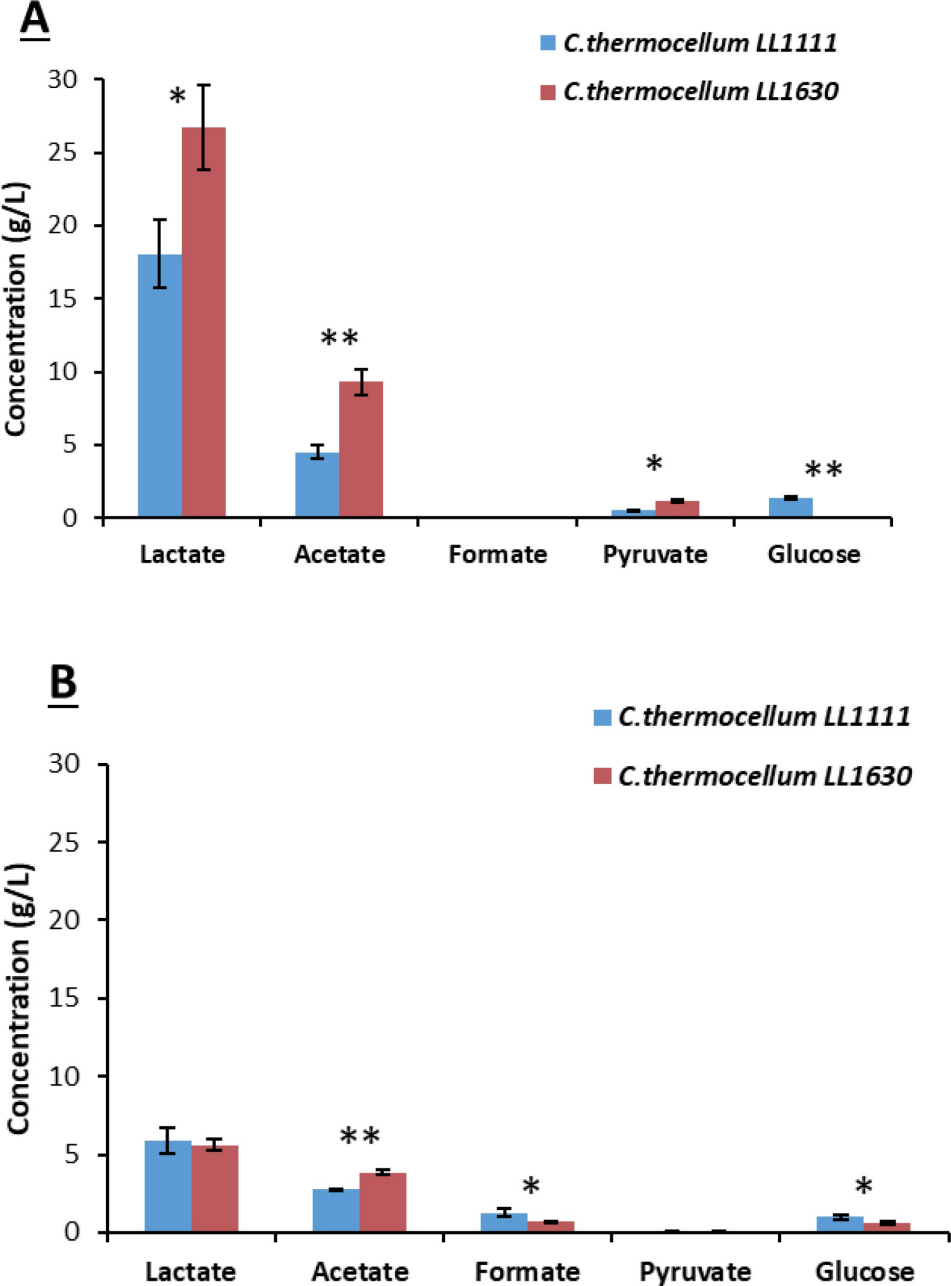
Final concentration of fermentation products with Avicel® (A) or S2 (B) (bottle fermentation with pH regulation) (* P value<0.05; ** p‐value<0.01).

The same cultures were repeated at a higher scale (50 mL) which allowed for monitoring pH changes in the growth medium to measure bacterial growth. During Avicel® fermentation, LL1111 growth resulted in a slower pH decrease compared to LL1630 (Figure S13), while the opposite was observed during growth in S2‐supplemented medium (Figure S14). Medium acidification essentially stopped (96 h) when the pH of the medium was 5.9–6.3 in Avicel® supplemented cultures, while the same happened at higher pH (6.3–6.8) for cultures growing on S2. As regards the final product concentration at t168 (Figure 11), apart from some small variations, the fermentation product profile observed in these cultures was comparable to that of tube fermentations (Figure 10). However, in S2‐supplemented cultures, *C. thermocellum* LL1630 proved to be more performing since it produced higher amounts of organic acids, which is LA (p‐value<0.05), acetate and formate (p‐value<0.01) compared to LL1111. More in detail, the final titer of LA for S2 fermentation was 3.77 g/L for LL1111 and 5.71 g/L for LL1630 (Figure 12). Therefore, these data confirm that *C. thermocellum* LL1630 should be a more suitable strain to ferment the S2 fraction from grape stalks.

For Avicel®‐supplemented cultures, it can be speculated that pH acidification was the main factor limiting complete substrate fermentation by both strain LL1111 and LL1630, since the pH of medium at the end of the bacterial growth was consistent with the limit for *C. thermocellum*.[^39^, ^84^] For *C. thermocellum* LL1630 cultures grown on S2‐supplemented medium, other factors seem to limit bacterial growth, since bacterial growth likely stopped at pH=6.8, which is significantly higher than *C. thermocellum* limit.

**Fermentation Tests at Regulated pH**. Maintaining the pH of the growth medium between 6 and 8 (Figure S15), dramatically improved the fermentation of Avicel® by both *C. thermocellum* strains LL1111 and LL1630 (Figure 12A). This confirms that, in not regulated pH conditions, *C. thermocellum* growth and metabolism on this substrate are mainly limited by pH decrease related to acid accumulation.^[39]^ In pH regulated cultures, a higher consumption of biomass by strains LL1111 and LL1630 and a lower accumulation of pyruvate and glucose resulted in a higher LA production as compared to not regulated cultures. More in detail, 18.04 g/L and 26.74 g/L LA were produced by strain LL1111 and LL1630, respectively. This seems related to the higher tolerance to LA of strain LL1630 (35 g/L) with respect to strain LL1111 (15 g/L).^[39]^ Eventually, pH regulation resulted in about 5 –7‐fold increase of LA titer with respect to not regulated cultures. It is worth noting that these are the highest LA titers reported for *C. thermocellum*, and the highest LA titers obtained through direct fermentation of a cellulosic feedstock by a single microbial strain to date.^[12]^

The same pH regulation approach was unable to improve fermentation of S2 by *C. thermocellum* strains to a similar extent (Figure 12B). LA production was limited to 5.6–5.8 g/L, which corresponds to an increase of 41 % for LL1111 and 17 % for LL1630 with respect to cultures without pH regulation. pH regulation did not promote significant increase of cellulose consumption, however, it reduced glucose and, especially, pyruvate accumulation (Figure 12B, Table 4)

**Table 4:** Product yield for S2 fermentation in pH regulated conditions.

C. thermocellum	Substr.	Yield (g/g)			
		Lactate	Acetate	Formate	Pyruvate
LL1111	S2	0.381±0.038	0.179±0.004	0.083±0.016	0.003±0.001
LL1630		0.366±0.023	0.250±0.008	0.044±0.005	0.004±0.001

These results confirm that the factor(s) that mainly limited *C. thermocellum* growth and fermentation on S2 was not medium acidification (Figure S16). Less efficient fermentation of complex lignocellulosic feedstocks with respect to pure cellulose has frequently been observed.[^84^, ^85^, ^86^, ^87^] A recent study reported decreased C. thermocellum growth and metabolism on co‐solvent enhanced lignocellulosic fractionation (CELF)‐pretreated poplar wood with respect to Avicel^®^.^[88]^ The authors speculated that this was related to the presence of residual amounts of xylan and lignin in pretreated wood. Evidence has been previously brought that lignin and soluble hemicellulose components inhibit C. thermocellum cellulosome activity^[89]^ and reduce *C. thermocellum* growth and solubilization of lignocellulosic substrates.^[90]^ Since S2 still contains a significant fraction of lignin and hemicellulose (Figure 9), it is likely that they contribute to inhibiting *C. thermocellum* fermentation with respect to cultures grown on Avicel^®^ in pH regulated conditions. Pretreatment optimization (that is slight reduction of xylan and lignin content) doubled short‐chain ester production by *C. thermocellum* from poplar wood.^[88]^ A similar further improvement of our cascade protocol seems therefore a promising strategy for enhancing fermentative production of LA by *C. thermocellum* from grape stalks.

**Process Outcomes**. In this study, a sustainable biorefinery process was developed and optimized to create a sustainable approach for utilizing all the components of grape stalks, aligning with a zero‐waste approach. The fractionation process yielded various components, including a polyphenolic fraction, two pectic fractions distinguished by varying degrees of esterification, lignin, and a cellulose‐enriched solid (S2).

The application of membrane treatment significantly minimized raw material waste and enhanced the quality of the liquid fractions through effective concentration and purification.

The cascade approach employed in this research integrates chemical and biological methods to exploit all components of grape stalks. This method begins with the extraction of bioactive compounds, followed by delignification, and culminates in the valorization of the cellulose‐rich fraction through biological conversion to produce lactic acid (LA) using engineered strains of *Clostridium thermocellum*.

Lactic acid (LA) is a high value product whose presence is already well established in the world market thanks to its countless applications in various fields such as bioplastics (e. g. polylactide), the food industry and the medical‐pharmaceutical industry.^[91]^

The polyphenolic fraction exhibited effective biocidal properties against several microorganisms known to cause wine spoilage. Combining these extracts with SO_2_ proved successful in preventing wine spoilage while reducing the necessary SO_2_ concentration for preservation, according to a circular approach, from wine waste to wine production. Furthermore, the deeper evaluation and exploitation of the phenols‐rich fraction for potential applications in the wine‐making industry as an effective biocide toward spoilage, complies with a circular economy approach, closing the loop by reuse in wine production. This step not only adds value diminishing the SO_2_ concentration necessary for preservation but also aligns with the Sustainable Development Goals (SDGs) by promoting the development of health‐beneficial products and supporting the reduction of food waste. Additionally, the study highlights the recovery and fractionation of pectin, revealing its significant antioxidant activity.

**Figure 13:**
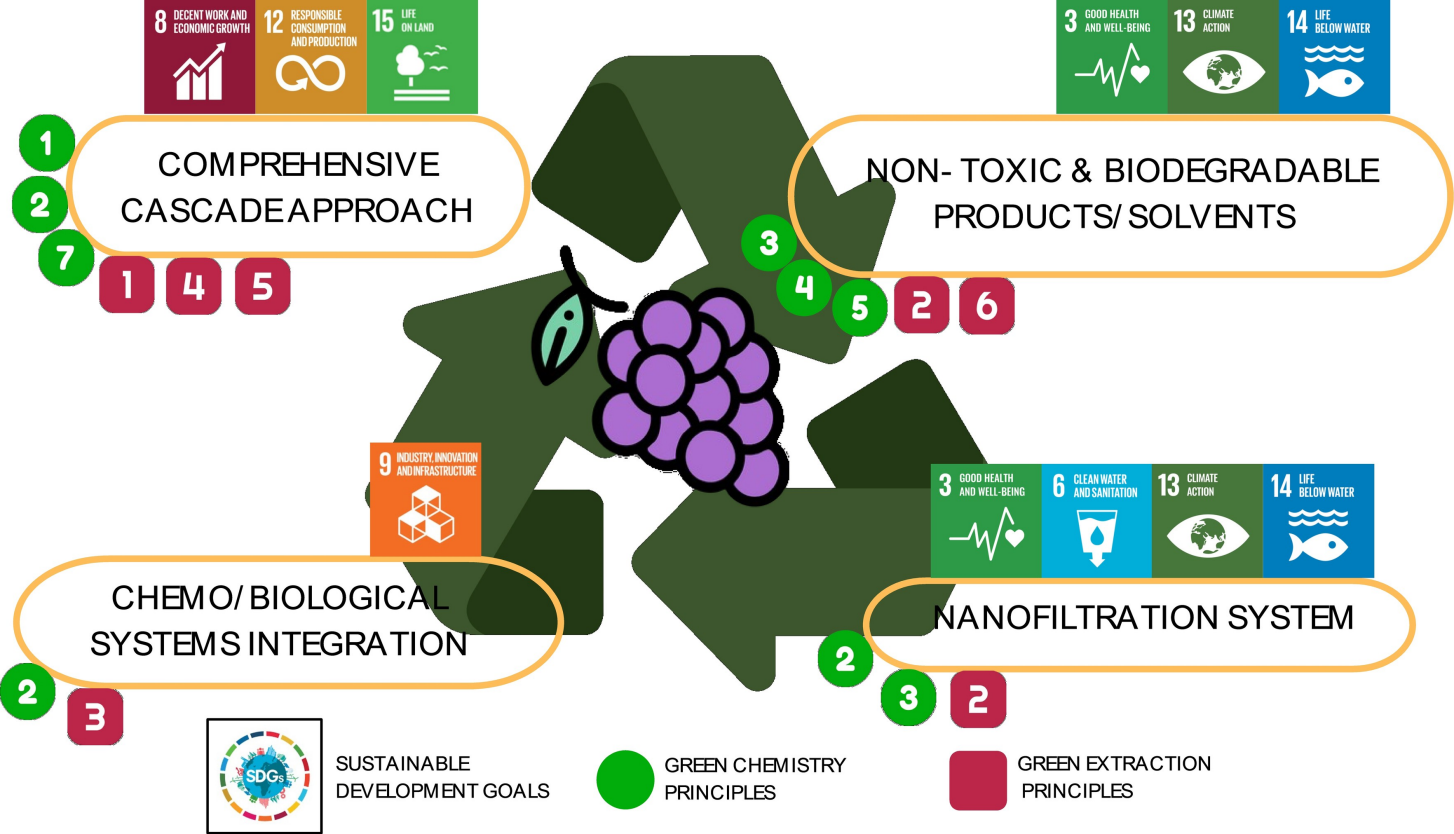
Evaluation of the whole grape stalk valorization process in terms of sustainability and green chemistry.

To minimize waste, the research incorporates a cost‐effective nanofiltration system to recycle large quantities of water and NaOH, significantly reducing the need for additional chemicals like sulfuric acid and ethanol. This method enhances the sustainability and economic viability of the process, supporting SDGs related to clean water and sanitation, responsible consumption, and production. The overall process traces the road to the development of a zero‐waste biorefinery (Figure 13).

## Conclusions

The findings from this study indicate that the valorization of grape stalks through this integrated biorefinery approach has potential applications in various industrial sectors, including cosmetics, nutraceuticals, and winemaking. By adopting advanced green extraction methods, eco‐friendly solvents, and biotechnological processes, this research sets a new standard for sustainable agricultural waste management.

This approach could represent a green step forward compared to the current state of the art in the valorization of by‐products from the wine industry. While previous studies focused on the extraction and valorization of single components (polyphenols,^[20]^ lignin.^[73]^..), this work aims to separate and use all of them as a resource to obtain value‐added products for different purposes, to minimize the by‐products.

This strategy is in accordance with the principles of Green Chemistry by promoting process intensification and resource efficiency.

The reduction, recycling and reuse of solvents and additives was also exploited, in view of the development of an overall zero‐waste biorefinery.

## Author Contributions

Carlotta Valle: Writing – original draft, Investigation, Formal analysis, Data curation. Giorgio Grillo: Writing – review & editing, Data curation, Methodology, Conceptualization. Emanuela Calcio Gaudino: Visualization, Methodology, Data curation. Paola Ponsetto: Investigation, Formal analysis, Data curation. Roberto Mazzoli: Methodology, Conceptualization, Investigation, Writing – review & editing. Giulia Bonavita: Formal analysis, Data curation. Pietro Vitale: Formal analysis, Data curation. Enrica Pessione: Methodology, Conceptualization, Investigation, Writing – review & editing. Emilia Garcia‐Moruno: Conceptualization, Investigation, Writing – review & editing. Antonella Costantini: Conceptualisation, Data Curation, Project administration, Funding acquisition. Giancarlo Cravotto: Supervision, Writing – review & editing. Silvia Tabasso: Writing – review & editing, Investigation, Supervision, Methodology, Conceptualization, Visualization.

## Conflict of Interests

There are no conflicts to declare.

## Supporting information

As a service to our authors and readers, this journal provides supporting information supplied by the authors. Such materials are peer reviewed and may be re‐organized for online delivery, but are not copy‐edited or typeset. Technical support issues arising from supporting information (other than missing files) should be addressed to the authors.

Supporting Information
