# Supplementary material for: Grape Stalks Valorization towards Circular Economy: A Cascade Biorefinery Strategy
Source: ChemSusChem. 2025 Feb 14;18(10):e202402536. doi: 10.1002/cssc.202402536 (PMC12094148; doi:10.1002/cssc.202402536)
Supplement: Supplementary file 1 — Supporting Information [file CSSC-18-e202402536-s001.pdf]

ChemSusChem

Supporting Information

Grape Stalks Valorization towards Circular Economy: A Cascade Biorefinery Strategy

Carlotta Valle, Giorgio Grillo,* Emanuela Calcio Gaudino, Paola Ponsetto, Roberto Mazzoli, Giulia Bonavita, Pietro Vitale, Enrica Pessione, Emilia Garcia-Moruno, Antonella Costantini, Giancarlo Cravotto, and Silvia Tabasso*

Grape Stalks Valorization Towards Circular Economy: A Cascade Biorefinery Strategy

Carlotta Valle,[a] Giorgio Grillo,[a*] Emanuela Calcio Gaudino,[a] Paola Ponsetto,[b] Roberto Mazzoli,[b] Giulia Bonavita,[a] Pietro Vitale,[a] Enrica Pessione,[b] Emilia Garcia-Moruno,[c] Antonella Costantini,[c] Giancarlo Cravotto,[a] Silvia Tabasso[a**]

[a] Department of Drug Science and Technology, University of Turin, Via P. Giuria 9, 10125 Turin, Italy.
[b]Department of Life Sciences and Systems Biology, University of Turin, Via Accademia Albertina 13, 10123 Turin, Italy.
[c]Council for Agricultural Research and Economics, Research Centre for Viticulture and Enology CREA-VE, Via P. Micca 35, 14100 Asti, Italy.
*Co-corresponding author: giorgio.grillo@unito.it
** Corresponding author: silvia.tabasso@unito.it

Supporting information

Table S1: MTC-5 medium composition.

Solution	Volume (mL)	Composition
A	45	Carbon source (Cellobiose, Avicel® or S2), Sodium MOPS salt $C_7H_{14}NNaO_4S$ in MilliQ water
B	2	Potassium citrate monohydrate ($C_6H_5O_7K_3 \cdot H_2O$), monohydrate citric acid ($C_6H_8O_7 \cdot H_2O$), sodium sulphate (Na_2SO_4), potassium dihydrogen phosphate (KH_2PO_4), sodium bicarbonate ($NaHCO_3$) in MilliQ water
C	1	Urea (CH_4N_2O), ammonium chloride (NH_4Cl) in MilliQ water
D	1	magnesium chloride ($MgCl_2 \cdot 6H_2O$), calcium chloride ($CaCl_2 \cdot H_2O$), iron (II) chloride ($FeCl_2 \cdot 4H_2O$), L-Cysteine hydrochloride monohydrate ($C_3H_7NO_2S \cdot HCl \cdot H_2O$), trace mineral (see F solution), MilliQ water
E	1	Pyridoxamine hydrochloride ($C_8H_{12}N_2O_2 \cdot 2HCl$), p-aminobenzoic acid ($C_7H_7NO_2$), d-biotin ($C_{10}H_{16}N_2O_3S$), vitamin B12 ($C_{63}H_{88}CoN_{14}O_{14}P$), MilliQ water
F	2.5 (in 50 mL of D)	manganese chloride ($MnCl_2 \cdot 4H_2O$), oxalyl chloride ($COCl_2 \cdot 6H_2O$), zinc chloride ($ZnCl_2$), copper chloride ($CuCl_2 \cdot 2H_2O$), boric acid (H_3BO_3), sodium molybdate ($Na_2MoO_4 \cdot 2H_2O$), nickel chloride ($NiCl_2 \cdot 6H_2O$) in MilliQ water

Fig. S1: Scheme of the cascade process with mass balance.

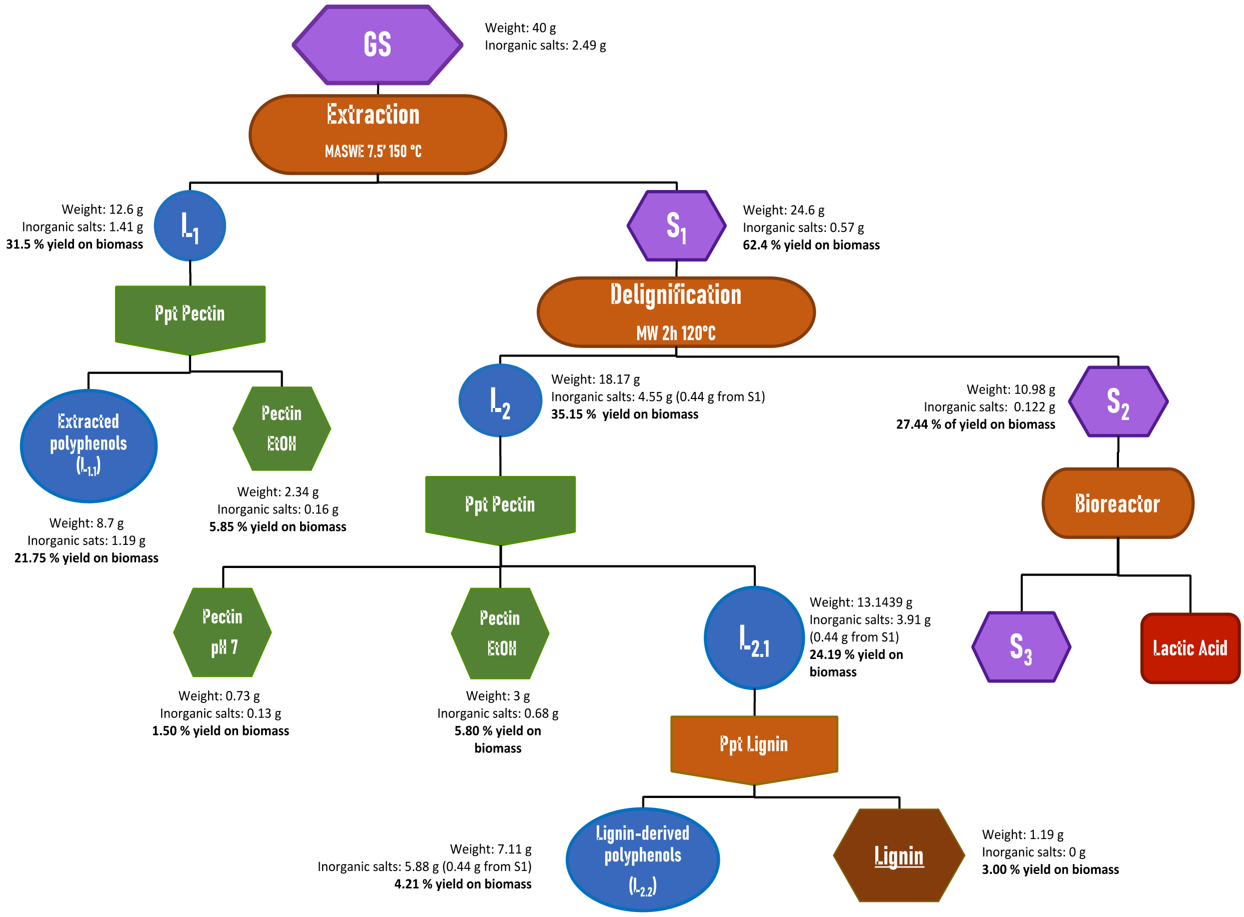

Table S2: Moscato must *characterization*.

**°Bx**	20
**Volatile acidity (g/L)**	0.07
**pH**	3.185
**Organic nitrogen (mg/L)**	84
**Inorganic nitrogen (mg/L)**	47

Table S3: Moscato wine characterization.

**Volatile acidity (g/L)**	0.52
**Alcohol (% v/v)**	11.53
**Acetaldehyde (mg/L)**	< 18
**SO_2 (mg/L)**	< 15 mg/L

Table S4: Analyses of the four different treatments after 3 months of conservation.

	No additives	S.D.	200 mg/mL SO_2	S.D.	100 mg/mL SO_2 + 5 mg /mL extracts	S.D.	5 mg/L extracts	S.D.
**Volatile acidity (g/L)**	1.085	0.035	0.935	0.021	1.005	0.007	> 1.2	-
**Acetaldehyde (mg/L)**	< 18	-	< 18	-	< 18	-	< 18	-
**SO_2 (mg/L)**	< 15	-	112.000	1.414	31.500	0.707	< 15	-
**Free SO_2 (mg/L)**	< 1	-	56.000	1.414	12.000	0.000	< 1	-
**IPT (GAE mg/L)**	146.500	2.828	159.000	1.414	377.000	24.042	308.500	20.506
**Tannins (g/L)**	< 0.3	-	< 0.3	-	< 0.3	-	< 0.3	-
**Catechins (mg/L)**	2.250	0.354	2.800	0.566	> 30	-	> 30	-
**Polyphenols (mg/L)**	< 150	-	< 150	-	913.500	130.815	737.500	27.577
**Abs 420**	0.128	0.010	0.094	0.000	0.697	0.262	0.637	0.003
**Abs 520**	0.055	0.009	0.031	0.005	0.311	0.150	0.256	0.000
**Abs 620**	0.036	0.004	0.021	0.001	0.122	0.106	0.056	0.005
**Intensity**	0.219	0.023	0.141	0.012	1.130	0.518	0.949	0.001
**Tonality**	2.388	0.235	3.122	0.508	2.302	0.262	2.489	0.008

Table S5: ANOVA and Post Hoc Tukey test results conducted on volatile acidity (A), IPT (B), catechins (C), polyphenols (D), Abs 420 nm (E), with the aim of comparation of the four different treatments.

**A**	**Mean - Volatile acidity (g/L)**	**Groups**		
**5 mg/L L1.1**	1.3	A		
**No Additives**	1.085		B	
**100 mg/mL SO_2 + 5 mg /mL L1.1**	1.005		B	C
**200 mg/mL SO_2**	0.935			C

**B**	**Mean - IPT (GAE mg/L)**	**Groups**		
**100 mg/mL SO_2 + 5 mg /mL L1.1**	377	A		
**5 mg/L L1.1**	308.5		B	
**200 mg/mL SO_2**	159			C
**No Additives**	146			C

**C**	**Mean - Catechins (mg/L)**	**Groups**	
**5 mg/L L1.1**	30	A	
**100 mg/mL SO_2 + 5 mg /mL L1.1**	30	A	
**200 mg/mL SO_2**	2.8		B
**No Additives**	2.25		B

**D**	**Mean - Polyphenols (mg/L)**	**Groups**	
**100 mg/mL SO_2 + 5 mg /mL L1.1**	913.5	A	
**5 mg/L L1.1**	737.5	A	
**No Additives**	140		B
**200 mg/mL SO_2**	140		B

**E**	**Mean - Abs 420**	**Groups**		
**100 mg/mL SO_2 + 5 mg /mL L1.1**	0.697	A		
**5 mg/L L1.1**	0.637	A	B	
**No Additives**	0.128		B	C
**200 mg/mL SO_2**	0.094			C

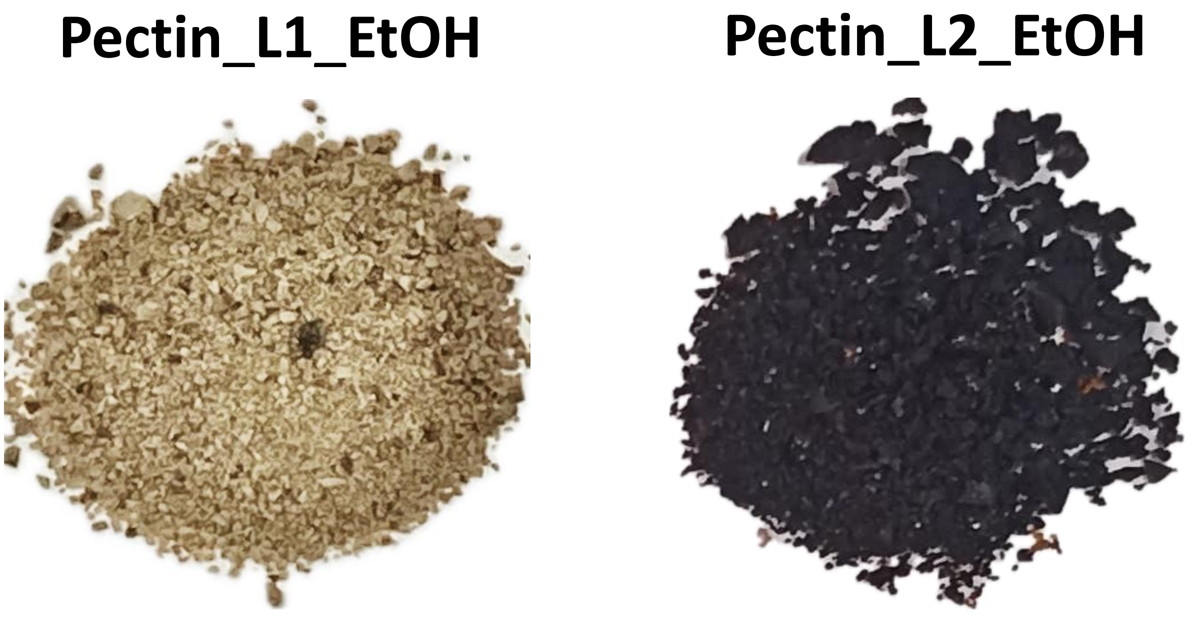

Fig. S2: Pectin precipitated with ethanol from L1 and L2.

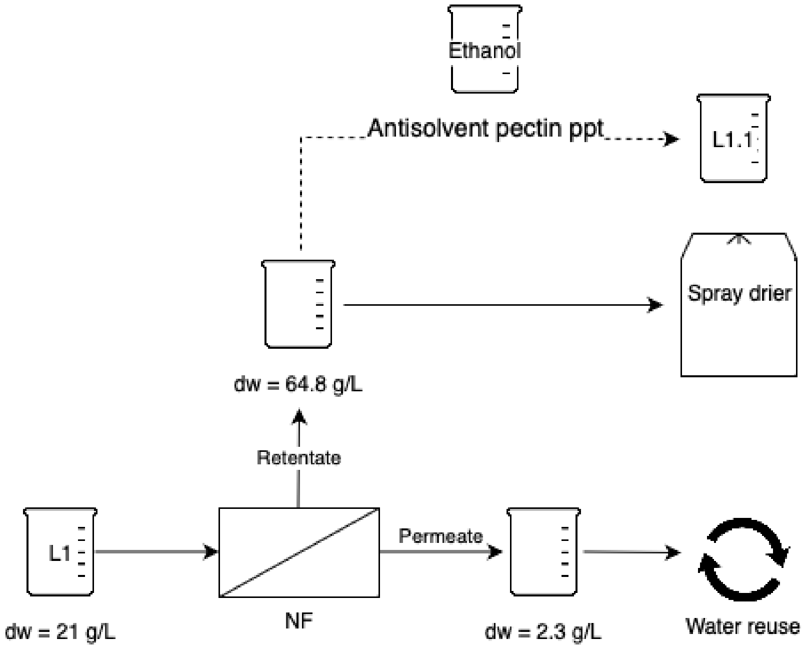

Fig. S3: Workflow of L1 membrane treatment process

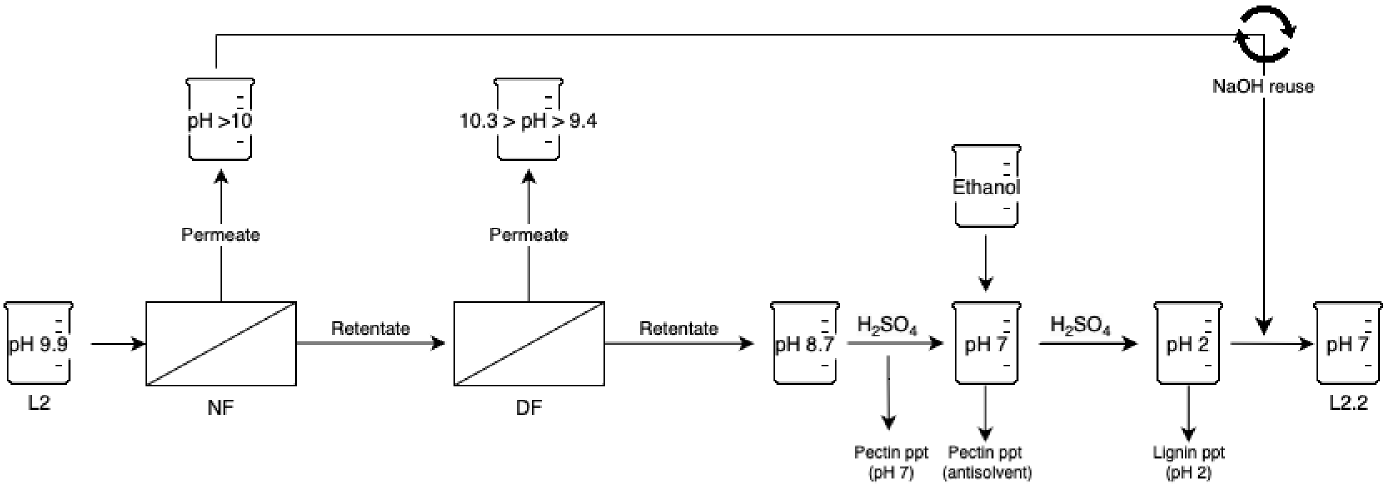

Fig. S4: Workflow of L2 membrane treatment process

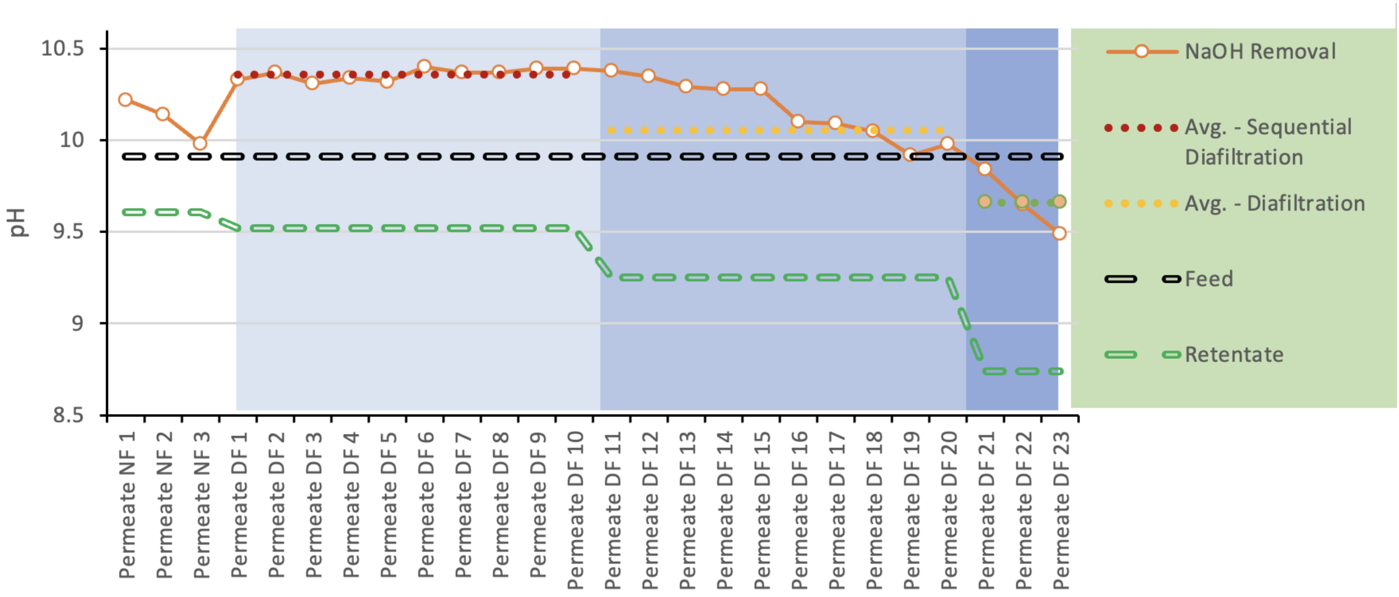

Fig. S5: Continuous NaOH diafiltration from L2

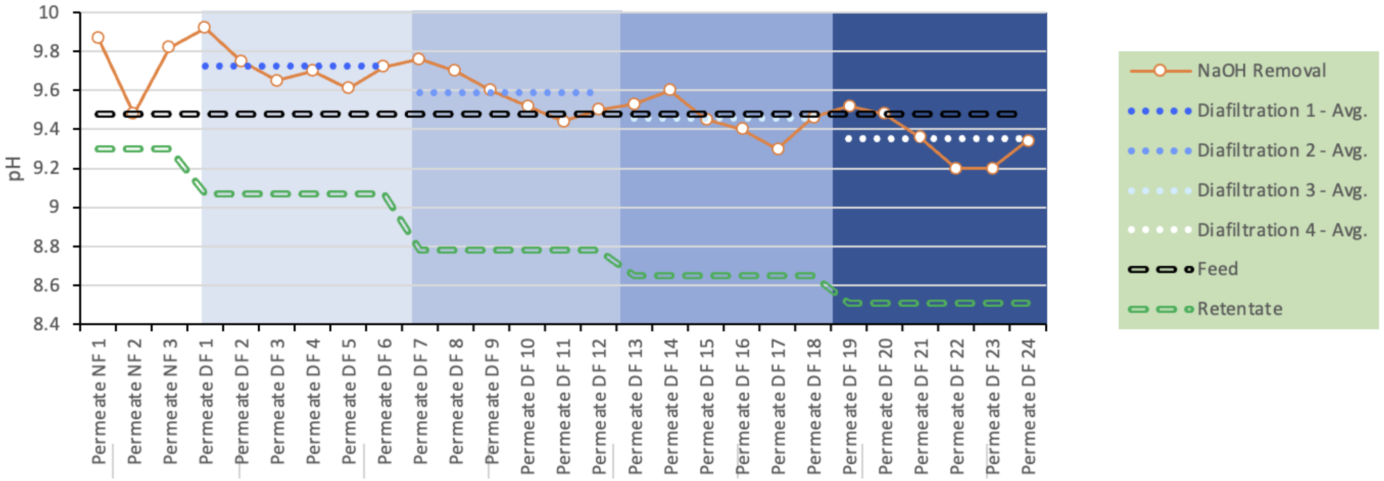

Fig. S6: Discontinuous NaOH diafiltration from L2

Table S6: A) Materials flow during the reuse and recycling of solvents and additives B) Reduction rates obtained in L2 downstream treatment

A	L1 nanofiltration			
Process	Solvents and Additives	NF process	Conventional	
Extraction	Extraction Water (L)	1.80	1.80	Upstream
NF	Recovered Water (L)	1.30	-	Downstream
	Volume of Retentate (L)	0.50	1.80	
Pectin precipitation	EtOH for pectin precipitation (L)	0.50	1.80	

	L2 membrane treatment			
Process	Solvents and Additives	NF process	Conventional	
Delignification	Water (L)	1.498		Upstream
	NaOH (g)	7.488		
	pH of L2	9.910		
	Residual NaOH in L2 (g)	4.84E-03		
NF+DF	NaOH recovery in Permeate (g)	4.80E-03	-	Downstream
	NaOH recovery in Permeate (%)	99.060	-	
	Volume of Retentate (L)	0.21	-	
	pH of Retentate	8.740	-	
	NaOH remaining in Retentate (g)	4.55E-05	-	
Pectin precipitation	H_2SO_4 addition to neutrality (g)	5.61E-05	0.006	
	EtOH for pectin precipitation (L)	0.208	1.498	
Lignin precipitation	Addition of alkaline permeate from ND+DF (L)	0.160	-	
	NaOH addition to neutrality (g)	-	0.596	
	Final Volume (L)	0.368	1.498	

B	Ratio	Reduction %
H_2SO_4 pH 7	106.50	99.06
EtOH	7.2	86.11
NaOH	-	100
Water	4.07	75.43

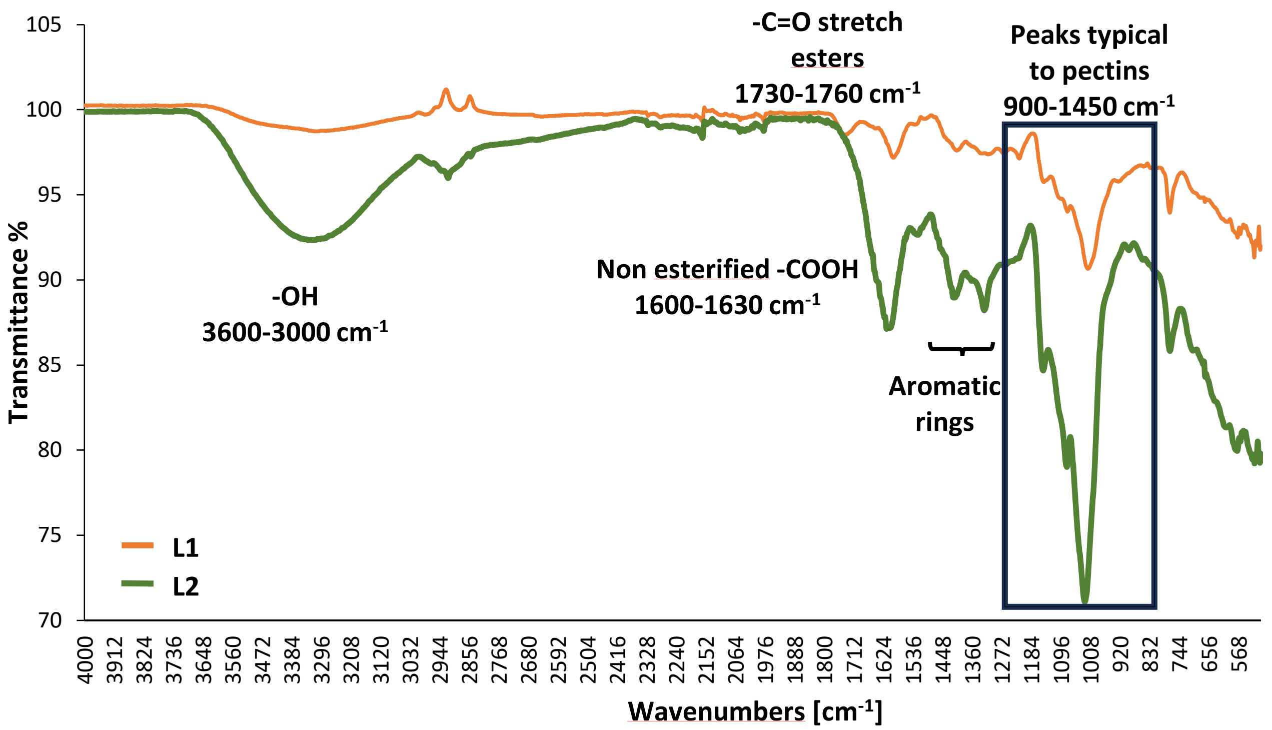

Fig. S7: ATR-FTIR of pectin precipitated from L1 and L2 with ethanol.

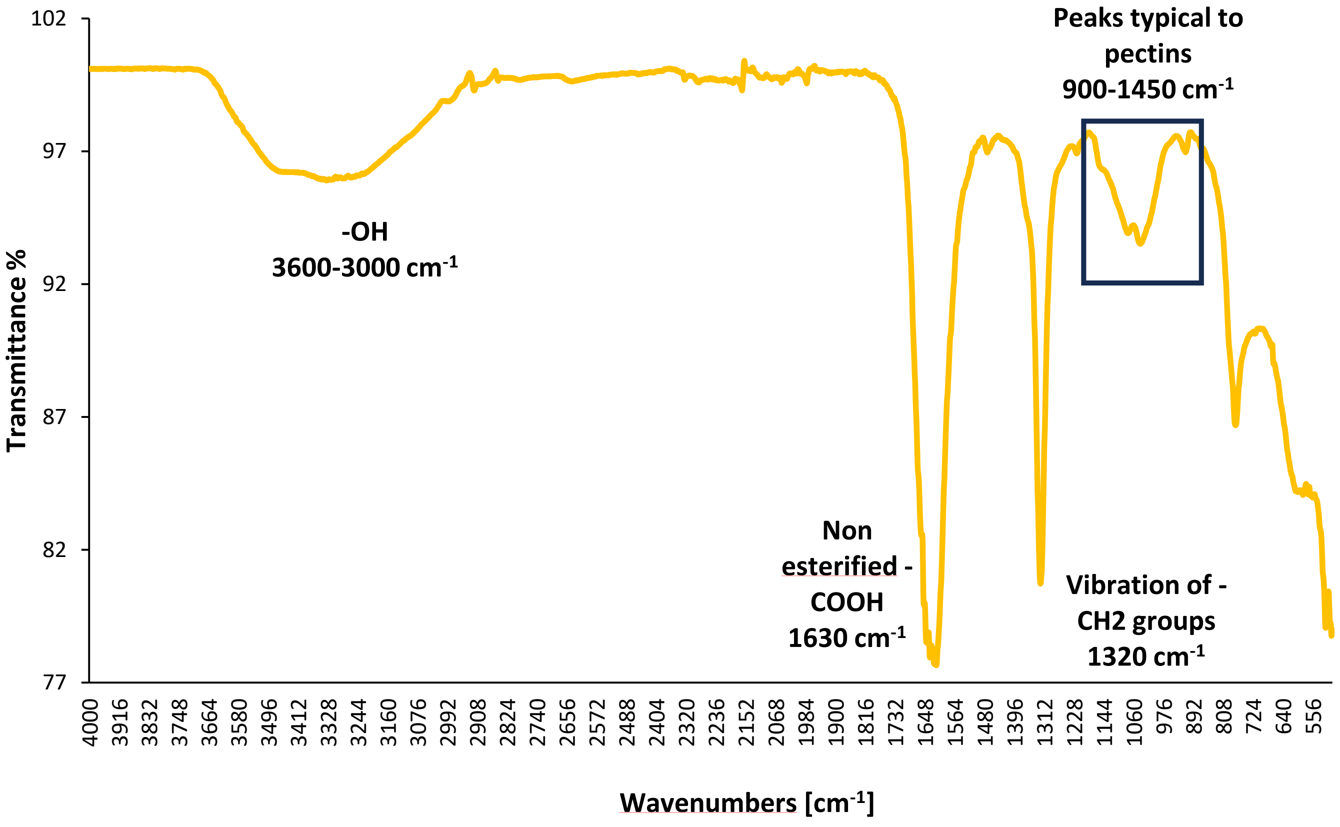

Fig. S8: ATR-FTIR of pectin precipitated at pH 7 from L2.

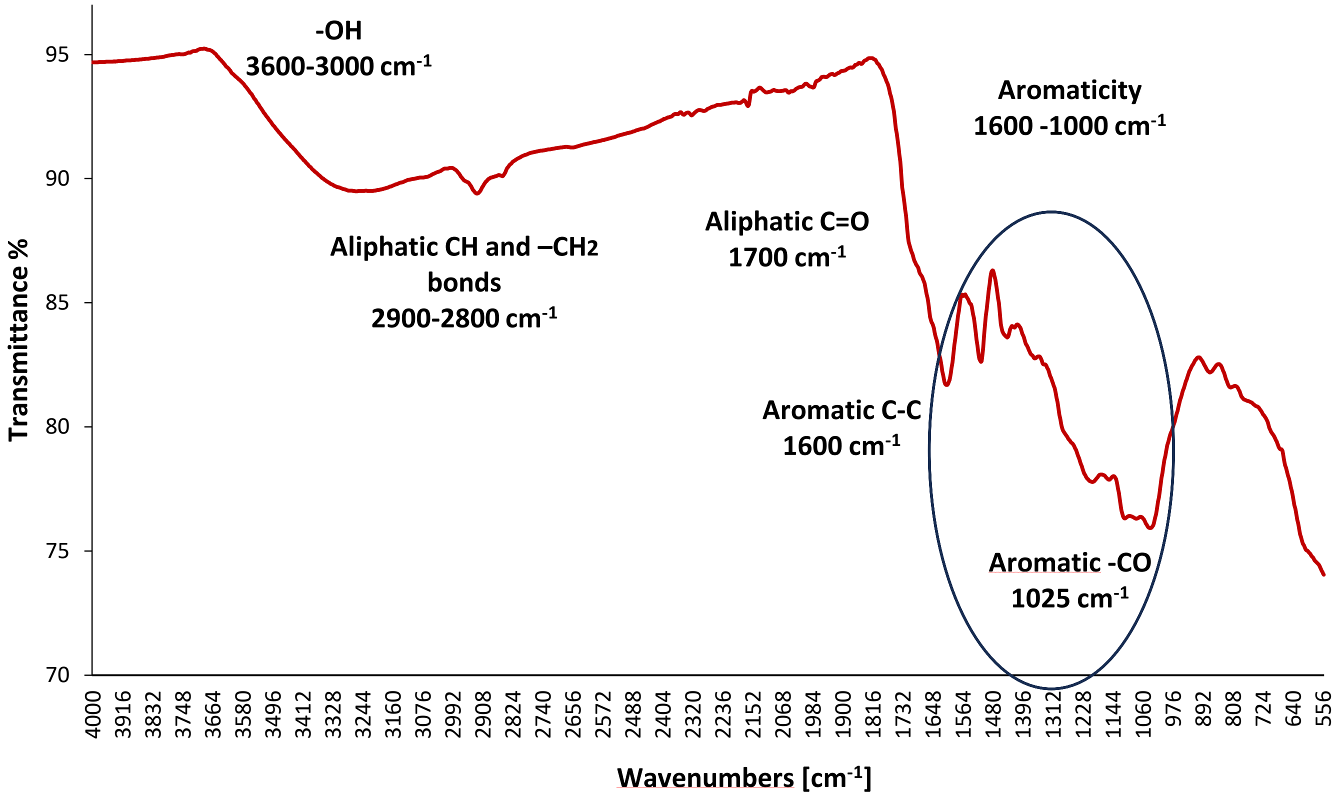

Fig. S9: ATR-FTIR of lignin fraction.

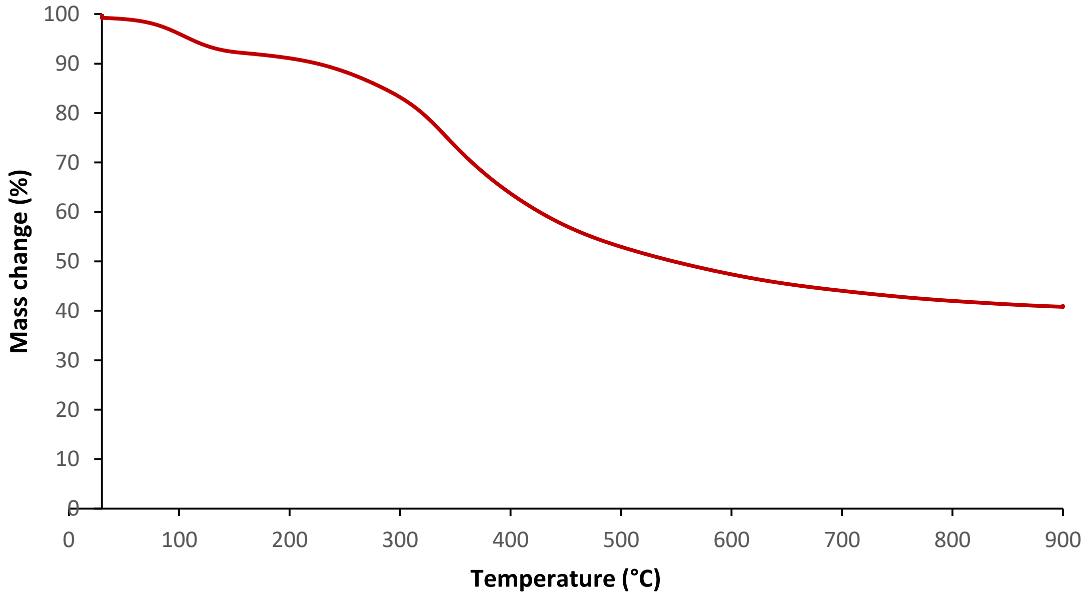

Fig. S10: TG analysis of lignin.

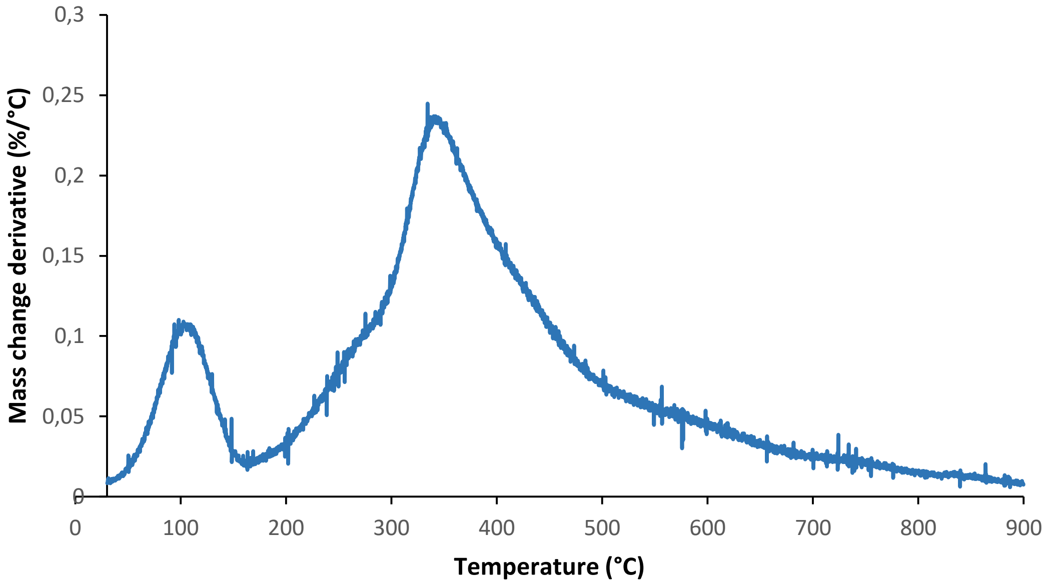

Fig. S11: DTG analysis of lignin.

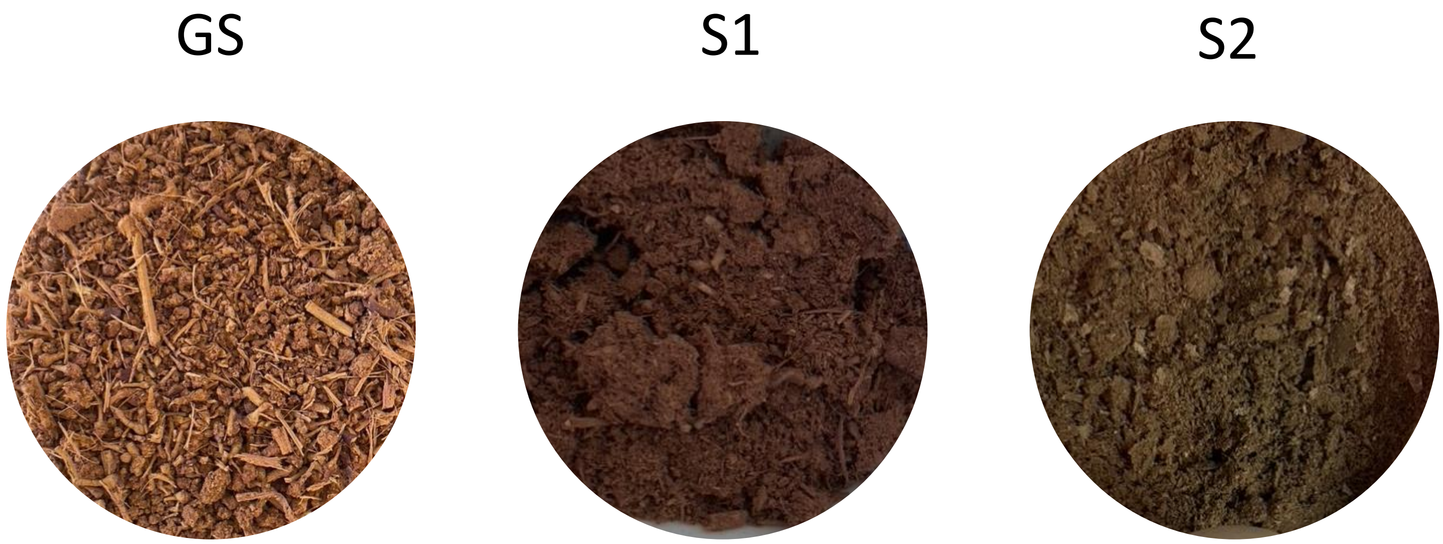

Fig. S12: Biomass fragmentation of GS during cascade process.

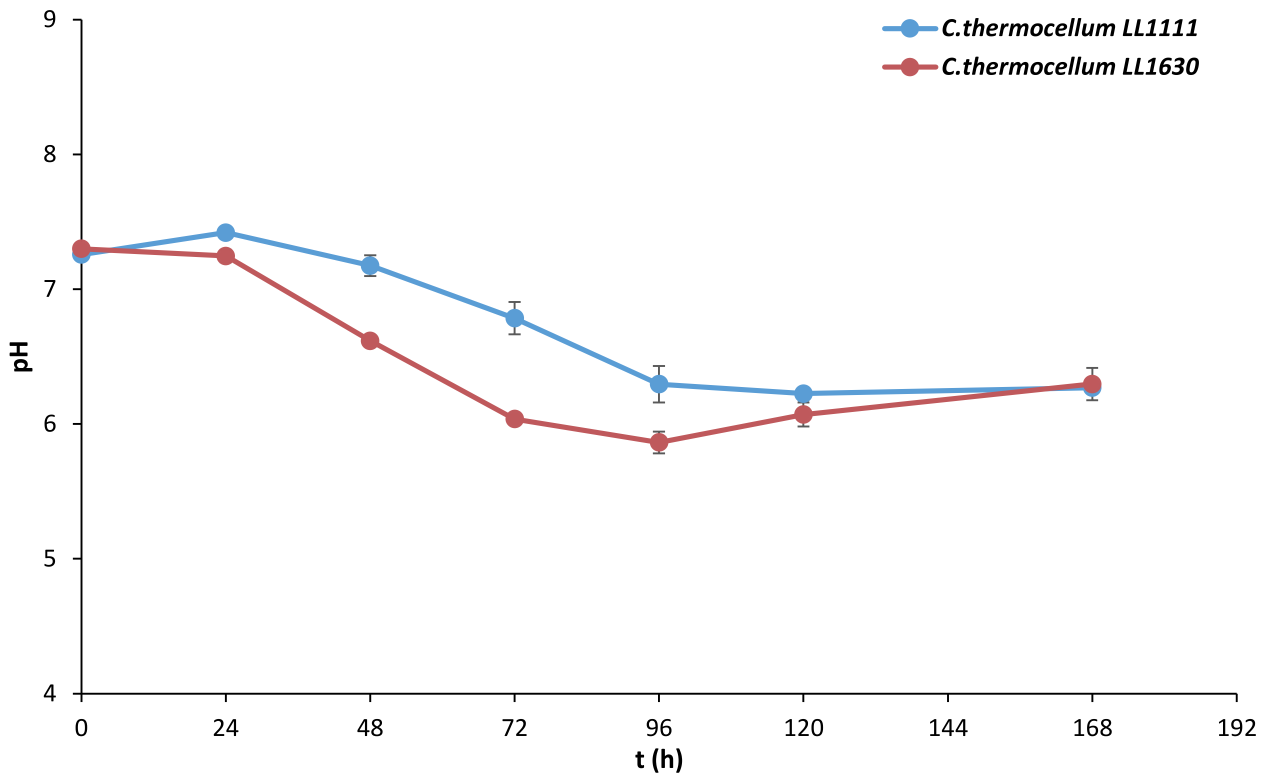

Fig. S13: pH evolution during flask fermentation of Avicel®.

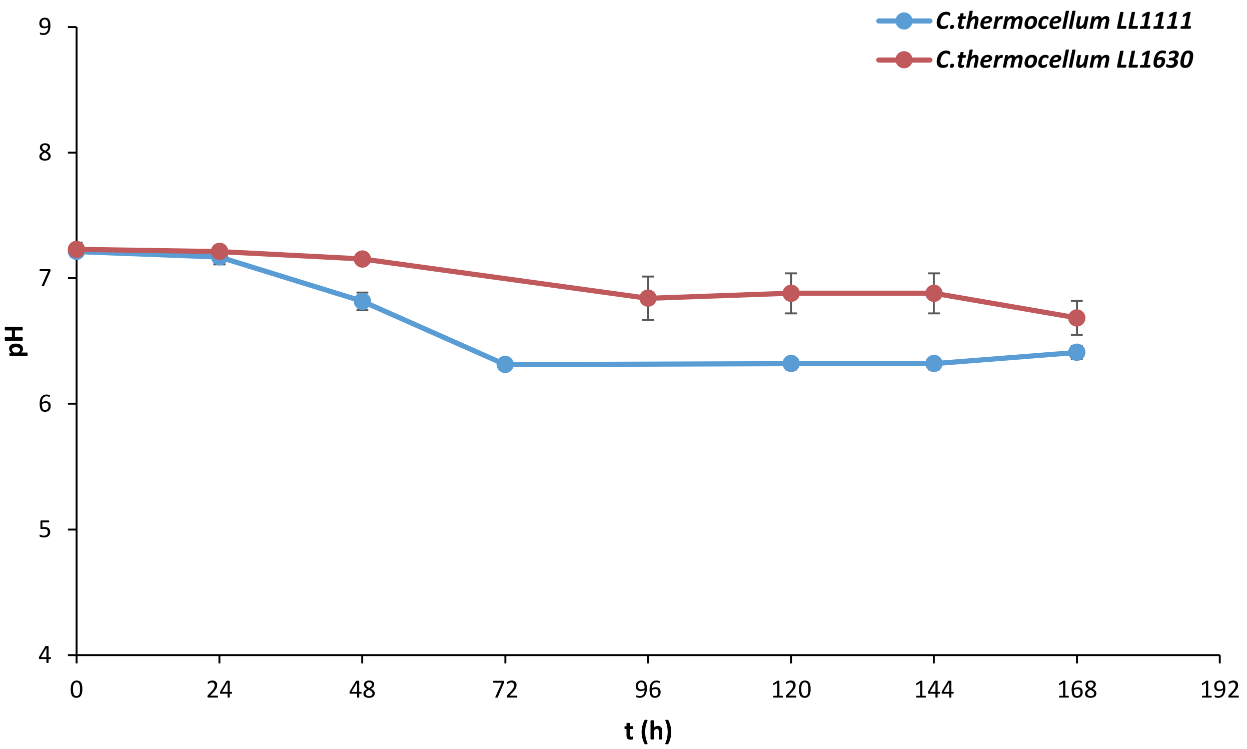

Fig. S14: pH evolution during flask fermentation of S2.

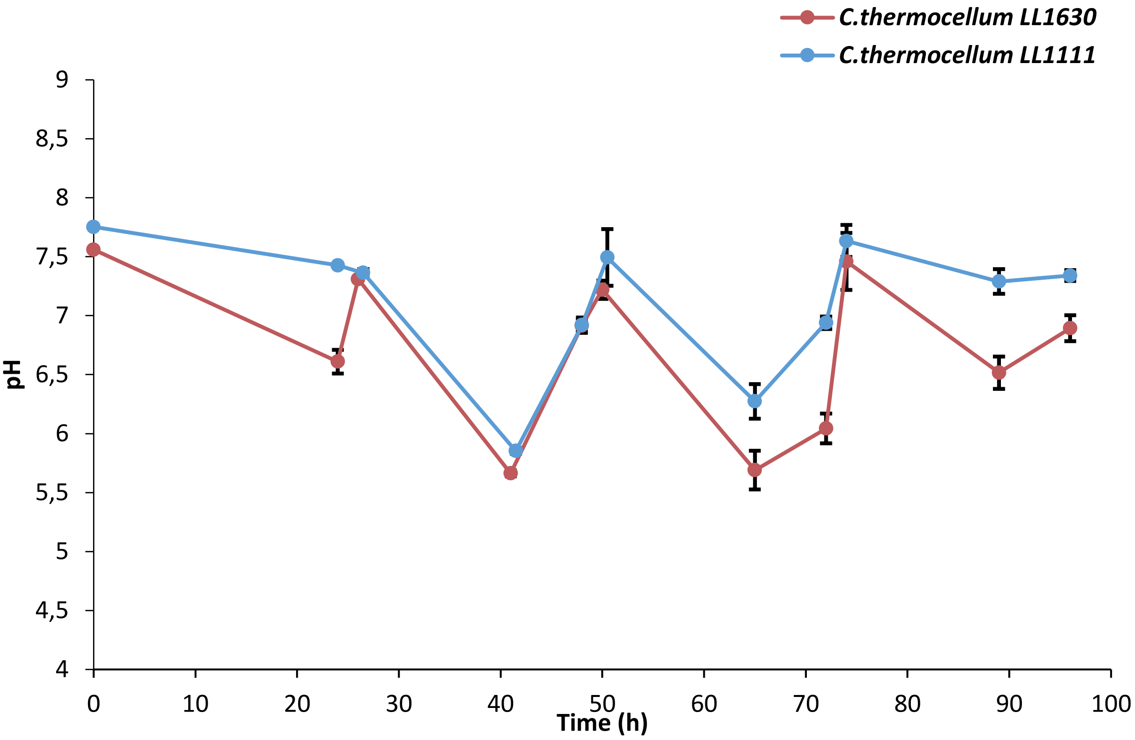

Fig. S15: pH evolution during bottle fermentation of Avicel® with manual pH regulation.

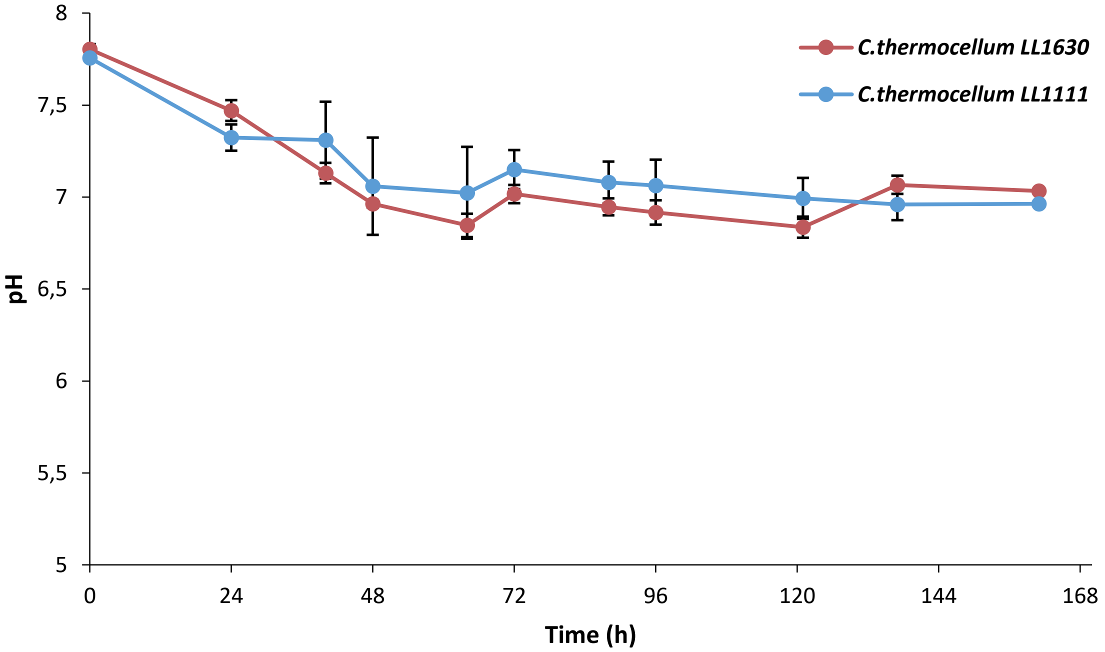

Fig. S16: pH evolution during bottle fermentation of S2 with manual pH regulation.
